# Insight into biological activities of chemically characterized extract from *Marrubium vulgare* L. *in vitro*, *in vivo* and *in silico* approaches

**DOI:** 10.3389/fchem.2023.1238346

**Published:** 2023-08-17

**Authors:** Aman Allah Gourich, Hanane Touijer, Aziz Drioiche, Ayoub Asbabou, Firdaous Remok, Soukaina Saidi, Farhan Siddique, Atika Ailli, Mohammed Bourhia, Ahmad Mohammad Salamatullah, Lahcen Ouahmane, Aicha Mouradi, Bruno Eto, Touriya Zair

**Affiliations:** ^1^ Laboratory of Innovative Materials and Biotechnology of Natural Resources, Research Team of Chemistry of Bioactive Molecules and the Environment, Faculty of Sciences, Moulay Ismaïl University, Meknes, Morocco; ^2^ Department of Pharmaceutical Chemistry, Faculty of Pharmacy, Bahauddin Zakariya University, Multan, Pakistan; ^3^ Department of Chemistry and Biochemistry, Faculty of Medicine and Pharmacy, Ibn Zohr University, Agadir, Morocco; ^4^ Department of Food Science and Nutrition, College of Food and Agricultural Sciences, King Saud University, Riyadh, Saudi Arabia; ^5^ Laboratory of Microbial Biotechnologies, Agrosciences and Environment (BioMAgE), Labeled Research Unit-CNRSTN 4, Cadi Ayyad University, Marrakech, Morocco; ^6^ Laboratoires TBC, Laboratory of Pharmacology, Pharmacokinetics and Clinical Pharmacy, Faculty of Pharmacy, University of Lille, Lille, France

**Keywords:** *Marrubium vulgare* L., polyphenols, LC/MS, antioxidant, toxicity, antimitotic, antimicrobial, antidiabetic

## Abstract

Aqueous extracts of *Marrubium vulgare* L. (*M. vulgare*) are widely used in traditional medicine for their therapeutic effects. Hence, this study aims to evaluate *in vitro*, *in vivo*, and *in silico* the biological activities of *M. vulgare* aqueous extract to further support their traditional use. Qualitative phytochemical tests of *M. vulgare* extracts showed the presence of primary and secondary metabolites, while quantitative analyses recorded revealed the contents of total phenols, flavonoids, and tannins, with values of 488.432 ± 7.825 mg/EAG gallic acid extract/g, 25.5326 ± 1.317 mg/EQ Quercetin extract/g and 23.966 ± 0.187 mg/EC catechin extract/g, respectively. Characterization of the phytochemical constituents of the extract revealed the presence of catechin and maleic acid as the most abundant while the evaluation of the antioxidant power revealed that the extract possesses significant antioxidant capacity, antimitotic potential, and antimicrobial properties against *Streptococcus agalactiae* and *Staphylococcus epidermidis* among many others. The antidiabetic activity of the extract showed a potent antihyperglycemic effect and a significant modulation of the pancreatic α-amylase activity as revealed by both *in vitro* and *in vivo* analysis, while an *in silico* evaluation showed that chemicals in the studied extract exhibited the aforementioned activities by targeting 1XO2 antimitotic protein, W93 antidiabetic protein and 1AJ6 antimicrobial protein, which revealed them as worthy of exploration in drug discovery odyssey. Conclusively, the result of this study demonstrates the numerous biological activities of *M. vulgare* and gives credence to their folkloric and traditional usage.

## 1 Introduction

For decades, medicinal herbs have been identified as a rich source of natural compounds that can be used to treat and prevent a wide range of illnesses (Bencheikh et al., 2021). According to the World Health Organization, 80% of people worldwide use traditional medicine including plants (Singh et al., 2018). Lesser side effects, efficacy, economic feasibility, and accessibility are the most important reasons for these practices (Ekor, 2014; Taib et al., 2020). Interestingly, the use of medicinal herbs in drug therapies has recently gained prominence, with about 25% of the total drugs used in developing countries being derived from plants (Hosseinzadeh and Nassiri-Asl, 2015; Alami Merrouni et al., 2021).

Noteworthy, many aromatic and medicinal plants have been identified as possessing an array of pharmacological activities such as antioxidant, anti-inflammatory, anticancer, antidiabetic, and antimicrobial activities, with these activities being majorly conferred by the secondary metabolites present in these plants (Yuan et al., 2016). Among the plethora of medicinal plants distributed across different geographical locations of the world, some belonging to certain families including the Lamiaceae family often stand out due to the rich pharmacological properties of plants from such families. Natively, the body’s antioxidant defense system is responsible for assuaging the effect of reactive oxygen species (ROS), however, the imbalance between the production of the enzymes that mediate the process and the production of ROS result in OS (Porto et al., 2013). Hence, the intake of exogenous antioxidants, particularly those from medicinal plants, is often recommended.

Diabetes, defined by persistent hyperglycemia, is a metabolic syndrome that develops when the pancreas either secrete insufficient insulin or the body no longer effectively utilizes secreted insulin (Deepthi et al., 2017; Sisodia and Sisodia, 2018). Interestingly, OS has also been reported to mediate the pathogenesis and progression of diabetes (Bouhrim et al., 2021; Ouassou et al., 2021). Hence, ameliorating OS is considered a therapeutic strategy for the treatment of diabetes (Dra et al., 2019). Similarly, the inhibition of enzymes that play pertinent roles in the metabolism of glucose is also a widely utilized approach for the management of type 2 diabetes. Notable among these enzymes are the human pancreatic alpha-amylase (HPA) which mediates the initial step involved in the catabolism of glucose by functioning in the hydrolysis of the glycosidic bonds present in starch. Currently, there exist several inhibitors of this enzyme in clinical usage, and they were reported to be efficacious. However, their use is often concomitant with several side effects including nausea, bloating, liver disorder, and weakness among many others (Bouyahya et al., 2021). Cancer remains a major health challenge globally, causing significant morbidity and mortality (Xiaomei and Herbert, 2006; Bencheikh et al., 2022b). Traditional treatment approaches like chemotherapy and surgery face limitations, including toxicity and drug resistance (Bencheikh et al., 2022b; Fakchich and Elachouri., 2021). Therefore, the search for novel drugs against cancer continues. Medicinal plants are a valuable resource, as they have contributed to the development of many clinically used drugs. Similarly, antimicrobial resistance has become a global public health concern, leading to increased morbidity, mortality, and healthcare costs. Acquired resistance mechanisms involve genetic mutations, horizontal gene transfer, and misuse of antimicrobial drugs (Onesti et al., 2015).

*Marrubium vulgare* L. is an annual or perennial herb from the Lamiaceae family and has been reported to possess an array of pharmacological properties including anti-inflammatory and hemostatic effects, as well as antihypertensive, sedative potential, and antimicrobial properties (Yousefi et al., 2014). Furthermore, it has also been reported to possess a panoply of bioactive compounds including premarrubiin, peregrinol, vulgatol, marrubenol, marrubiol, verbascoside, and forsythoside B among many others (Karryev et al., 1976; Pukalskas et al., 2012; Yousefi et al., 2013).

As a result of the multi-pharmacological properties of this plant, it has emerged as an attractive option for exploration for the discovery of phyto-compounds that can be used in the treatment of a wide range of diseases and disease-inducing conditions. Prominent among the biological phenomena that drive diseases is oxidative stress (OS). Notably, OS has been identified as a driver of several diseases, including diabetes, Alzheimer’s disease, Parkinson’s disease, cancer, and cardiovascular diseases (Ladoh Yemeda et al., 2014; Loizzo et al., 2017).

*M. vulgare* L. has been reported to possess antioxidant properties in folkloric use, and the antioxidant power of a plant often varies based on the source of the plant. Medicinal plants, such as *M. vulgare* L.*,* are being extensively studied for their bioactive compounds, as they offer potential as inhibitors and therapeutic agents. Developing alternatives to existing drugs is crucial in combating antimicrobial resistance, and medicinal plant compounds are being explored in this quest (Djahra, A. B. et al., 2013).

Therefore, this study aims to investigate the antioxidant, antimitotic, antimicrobial, and antidiabetic activities of *M. vulgare* leaves from Morocco. Additionally, the study aims to characterize the phytochemical constituents present in these leaves. By examining these properties and compounds, we aim to contribute to the ongoing search for effective treatments for cancer and antimicrobial-resistant infections.

## 2 Materials and methods

### 2.1 Harvest of *M. vulgare* leaves

**FIGURE 1:**
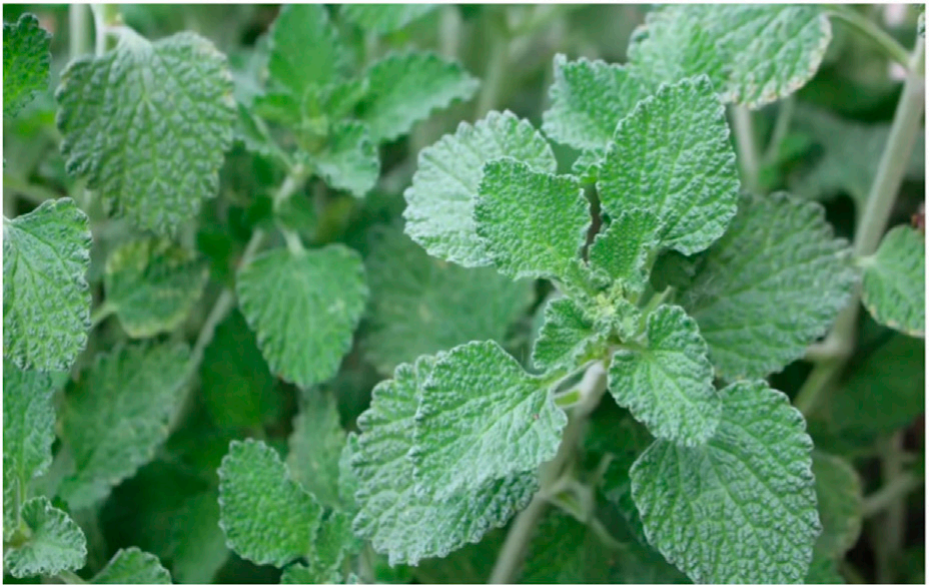
Morphological appearance of the *M. vulgare* during harvest (April 2020, Region Fez Meknes, Morrocco).

The plant material utilized in this study consists of the leaves of *M. vulgare* and they were harvested from the region of Fez Meknes (Moulay Idriss Zerhoun) in April 2020 during the flowering period of the plant. Subsequently, the plant samples were carefully placed in ventilated bags and subjected to a thorough cleaning to eliminate any potential contaminants or debris that could compromise the purity of the plant samples, after which its identity was authenticated at the Department of Botany of the Scientific Institute of Rabat. Furthermore, the plant samples were subjected to a drying process to remove excess moisture; this drying was carried out in a well-ventilated room and away from sunlight at a temperature range of 30°C–40°C for 10 and 15 days. Table 1 presents information on the region where the plant was harvested while Figure 1 depicts Morphological appearance of the harvested plant.

**TABLE 1:** Details of the location of *M. vulgare* plants used in this study.

Latin name	Harvest site			Parts used	Harvest year	Latitude (x)	Longitude (y)	Altitude (m)
	City	Region	Locality					
*M. Vulgare*	Meknes	Molay driss zerhoun	Madchar al Aama	Leaves	15/02/2020	5°31′06″W	34°03′21″ N	566

### 2.2 Quality control of plant material

#### 2.2.1. Dry matter moisture content

Briefly, 5 g of the dried leaves were weighed into a pre-dried and tared crucible, after which the crucibles were placed into an oven at 103°C for 24 h. Subsequently, the crucibles were allowed to cool in a desiccator and were re-weighed to determine the final mass. This analysis was conducted in accordance with the guidelines outlined in the French association of standardization (AFNOR) standard (NF - V03-402 1985) (AFNOR, 1985). The results obtained from the analysis were expressed as a percentage of the dry matter content using the formula below:

$$TH\%=\left(\frac{M_{0}-M_{1}}{M_{0}}\right)\times 100$$

Where:

**TH%**: humidity level

**M**_**0**_ **:** initial mass of the plant leaves;

**M**_**1**_ **:** mass obtained after drying.

#### 2.2.2 pH determination

To determine the pH of the leaves, 2 g of the leaves sample was placed in 10 ml of hot-filtered water. The resulting solution was purified by filtering and was allowed to undergo cooling. Subsequently, the electrode of a pH meter was immersed in the filtrate to determine its pH value.

#### 2.2.3 Titratable acidity

The titratable acidity of the leaf samples, which represents the total acidity of a solution and encompasses the free minerals and organic acids, was determined by weighing 10 g of the powdered leaf samples into 50 ml of boiling distilled water and was subjected to stirring for 15 min. Subsequently, the volume of the resulting solution was adjusted to 100 ml using filtered water and was thereafter filtered. The filtrate was subjected to titration using a 0.01 N NaOH solution, with the addition of a few drops of phenolphthalein indicator. Noteworthy, the titration process was continued until a noticeable color change signified by the presence of a persistent pink hue occurred (Bergeron, 1995). The titer values were converted into its citric acid equivalent using the conversion factor below:

$$A\%=Dilution\;factor*\;\frac{Weight\;of\;equivalent.acid*Normality\;of\;NaOH*titration\;volume\;\left(ml\right)}{Sample\;weight\;\left(g\right)}$$

#### 2.2.4 Ash content (NFV05-113, 1972)

The method employed in this study was based on the calcination of the leaf samples in a muffle furnace at a temperature of 550°C until white ashes of constant weight were achieved in accordance with AFNOR standard (AFNOR, 1972). The organic matter content was quantified according to the equation below:

$$MO\%=\left(\frac{W_{1}-W_{2}}{TS}\right)\times 100$$

Where

MO%: Organic material

**W**_**1**_ **:** Weight of capsule and sample before calcination

**W**_**2**_ **:** Weight of capsule and sample after calcination

**TS:** Test simple

Following the determination of the organic matter content, the ash content was determined as follows using the equation below:

Ash %=100−OM %

Ash %: Ash content

#### 2.2.5 Heavy metal assays: atomic emission spectrometry coupled with induced plasma (ICP-AES)

The leaf samples were investigated for the presence of heavy metals including arsenic (As), cadmium (Cd), chromium (Cr), iron (Fe), lead (Pb), antimony (Sb), and titanium (Ti). Noteworthy, there exists a specific contamination limit standard, which serves as a regulatory guideline, however, some exceptions are provided for medications whose primary ingredients have been established to accumulate significant levels of Cd. To determine the levels of the heavy metals, aqua regia (HNO_3_ + 3HCl) and the normalized mineralization, which helps to circumvent issues related to sample representativeness, were employed in accordance with the AFNOR guideline. To perform the analysis, aqua regia was firstly prepared by mixing 1 mL of nitric acid (HNO_3_; 99%) with 2 ml of hydrochloric acid (HCl; 37%) and heated to a temperature of 200°C, after which it was allowed to cool for 2 hours. Subsequently, the supernatant was carefully extracted and filtered through a 0.45 µm membrane filter. To ensure accuracy, the filtrate is then supplemented with 15 mL of distilled water. Following the preparation of the aqua regia, 0.1 g of the crushed plant leaves was combined with 3 mL of the prepared aqua regia, after which the concentration of the heavy metals was determined by using the inductively coupled plasma atomic emission spectroscopy (ICP-AES) utilizing the Ultima 2 Jobin Yvon instrument in accordance with protocols established in Özcan (Musa Özcan, 2006).

### 2.2 Microbial material

The antimicrobial activity of the plant extract was evaluated against nine bacterial strains and seven fungal strains known for their high resistance, invasiveness, and pathogenic properties. The bacterial strains are *Staphylococcus epidermidis*, *Enterobacter cloacae*, *Streptococcus agalactiae* (B), *Klebsiella pneumoniae*, wild *Escherichia coli*, ESBL *Escherichia coli*, *Proteus mirabilis*, *Pseudomonas aeruginosa,* and *Staphylococcus aureus* BLACT, while the fungal strains included *Candida albicans*, *Candida krusei*, *Candida tropicalis*, *Candida parapsilosis*, *Candida dubliniensis*, *Saccharomyces cerevisiae* and *Aspergillus niger*. Noteworthy, these microorganisms were obtained from the Mohamed V-Meknes Provincial Hospital and were initially stored in −80°C 20% glycerol stock. Before the utilization of the microorganisms, they were subcultured and grown on Mueller Hinton agar and Sabouraud agar for the bacterial and fungal strains, respectively.

### 2.4 Aqueous extract preparation of *M. vulgare*

30 g of powdered *M. vulgare* leaves was introduced into a 1-liter Erlenmeyer flask containing 600 mL of purified water. The mixture obtained was subjected to heat at 70°C for 1 h, after which it was filtered. Subsequently, the filtrate was subjected to an evaporation process under reduced pressure after cooling at room temperature. The residue obtained after the cooling was oven-dried at 50°C and subsequently weighed to calculate the extraction yield (Chavan et al., 2001).

### 2.5 Qualitative screening of phytochemicals

To determine the phytochemicals present in *M. vulgare* leaves, various solvents including water, chloroform, methanol, and petroleum ether were utilized. To determine the presence of alkaloids, Dragendorff and Mayer reagents were employed, while Hydrochloric acid and isoamyl alcohol were used to detect the presence of catechin tannins, and tiasny’s reagent, sodium acetate, and ferric chloride were used to characterize gallic tannins. Also, the presence of sterols and triterpenes was detected using acetic anhydride and strong sulfuric acid, Magnesium chips, isoamyl alcohol, and diluted hydrochloric alcohol were used to detect the presence of flavonoids, while chloroform, dilute ammonia, and hydrochloric acid was used for the detection of quinone substances (Tamert et al., 2017).

### 2.6 Quantification of polyphenols content

To determine the number of total polyphenols in the *M. vulgare* extract, the Folin-Ciocalteu reagent was employed. 500 µL of freshly prepared 0.1X Folin-Ciocalteu reagent and 2 mL of sodium carbonate solution (20% Na_2_CO_3_) were mixed with 100 µL of the extract. The resulting mixture was incubated for 30 min at room temperature and the absorbance was measured at a wavelength of 760 nm with referce to a standard (Ali-Rachedi et al., 2018).

### 2.7 Quantification of flavonoid content

The quantification of the flavonoid content of *M. vulgare* extract was conducted using the AlCl_3_ method, as described by Barros et al. (Barros et al., 2011). To perform the quantification, 2 μL of the extract was mixed with 10 μL of 10% aluminum chloride solution and 2 mL of distilled water, after which 3 mL of absolute methanol was added to the mixture. Subsequently, the mixture was incubated for 2 hours in the dark, and the absorbance was measured at 433 nm.

### 2.8 Quantification of tannins content

50 µL of *M. vulgare* leaves extract was added to 1,500 µL of 4% vanillin/methanol workaround and subjected to vigorous mixing. Subsequently, 750 µL of concentrated HCl was added to the mixture, and the resulting mixture was allowed to react at room temperature for 20 min. Ultimately, the absorbance of the mixture formed after the reaction was measured at 550 nm concerning a blank (Lorrain et al., 2011).

### 2.9 HPLC/UV-ESI-MS analysis of *M. vulgare*

The phytochemicals present in the *M. vulgare* extract were chromatographically characterized using high-performance liquid chromatography (HPLC) by employing an UltiMate 3000 system. Notably, the system contained a reverse-phase C18 column (250 mm × 4 mm, id 5 μm, Lichro CART, Lichrospher, Germany) with a sample changer, and the tests were conducted at a maintained temperature of 5°C. The temperature of the column was maintained at 40°C, while the mobile phase consisted of ultrasonically degassed solutions of 0.1% formic acid in water and 0.1% formic acid in acetonitrile and possessed gradient composition as follows: starting with 2% B at 0 min, followed by a linear change to 30% B at 20 min, 95% B at 25 min, and returning to 2% B at 26 and 30 min. Furthermore, the flow rate was set at 1 ml/min, and the injection volume was 20 µL. Detection of the compounds was performed using a Maxis Impact HD in MS/MS mode with negative electrospray ionization, while broadband collision-induced dissociation was employed for fragmentation. Additionally, UV detection was carried out using an L-2455 diode array detector, scanning in the wavelength range of 190–600 nm. Specific acquisition wavelengths of 280, 320, and 360 nm were utilized for analysis. Various parameter values were set for optimal performance, including a capillary voltage of 3000 V, the drying gas temperature of 200°C, dry gas flow rate of 8 L/min, vaporizing gas pressure of 2 bar, and an offset plate of −500 V. Nitrogen was used as the nebulization and desolvation gas, and mass spectrometry data were collected within the range of 50–750 m/z. Data acquisition and evaluation were performed using the Sysremsoftware program, specifically designed for chromatography data analysis in the Thermo ScientificTM ChromeleonTM 7.2 platform.

### 2.10 Antioxidant activities

#### 2.10.1 Free radical scavenging test

The free radical scavenging ability of the plant extract was determined using (2,2-diphenyl-1-picrylhydrazyl) assay protocol as detailed in (Bencheikh, et al., 2022a). To perform this assay, 200 µL of the extract at varying concentrations were placed in mixed with 2.8 mL of an ethanolic solvent containing DPPH (2.4 mg/100 mL) with an estimated absorbance of 0.6 and 0.7 at 517 nm. Subsequently, the resulting mixture was incubated for 30 min at ambient temperature and the absorbance was measured at 517 nm. To serve as positive controls for the experiment, solutions of ascorbic acid and butylhydroxyanisole (BHA) at the same concentrations as the plant extract was prepared while a blank experiment was also carried out using absolute ethanol. The results of the analysis were expressed as the percentage reduction in DPPH* (AA %) according to the equation below:

$$AA\%=\frac{A_{Control}-A_{Sample}}{A_{Control}}\times 100$$ (4)

Where:

**AA%**: Inhibition percentage;

**A**_**Control**_: Absorbance of the mixture, which contains only the radical DPPH solution;

**A**_**Sample**_: Absorbance of the samples to be assessed solution in the existence of DPPH; The relationship between the concentration of the plant extract and the percentage modification of the antioxidant activity (percentage of inhibition) was plotted on a graph from which the IC_50_ was determined.

#### 2.10.2 Ferric-reducing antioxidant power (FRAP)

The Iron reduction ability of *M. vulgare* extract was investigated using FRAP assay using the protocol described by Zovko et al. (Koncic et al., 2010). To perform the analysis, 2.5 mL of 1% potassium ferricyanide [K3Fe(CN)_6_] solution and 2.5 mL of phosphate buffer solution (pH = 6.6) were mixed with 0.5 mL of varying concentrations of the extract (same concentrations used in the DPPH test). The resulting mixtures were incubated in a water bath at 50°C for 20 min, after which the reaction was terminated by adding 2.5 mL of 10% trichloroacetic acid. Subsequently, the mixture was subjected to centrifugation at 3,000 revolutions per minute for 10 min. Aliquots from each concentration previously utilized were mixed with 2.5 mL of purified water and 0.5 mL of 0.1% FeCl_3_ solution. The absorbance of the mixtures was measured at 700 nm against a blank solution prepared using the same protocol, with distilled water replacing the plant extract. The calibration of the spectrophotometer was done using the blank solution, while the absorbance of standards including ascorbic acid, BHA, and BHT was also measured and compared.

#### 2.10.3 Total antioxidant capacity (TAC)

The TAC of the extract was determined by evaluating its ability to reduce molybdenum (VI) to molybdenum (V) via a reaction that leads to the formation of a green-colored phosphate/Mo(V) complex under acidic pH conditions. To perform this assay, 100 μL of *M. vulgare* decoction was mixed with 3 ml of a solution containing sulfuric acid (0.6 N), sodium phosphate (28 mM), and ammonium molybdate (4 mM). The tubes containing the mixture were then incubated at 95°C for 90 min and were allowed to cool to room temperature. Subsequently, the absorbance was measured at 695 nm against a blank that contains only the reaction mixture without the plant extract. The TAC of the extract was expressed in milligrams of ascorbic acid equivalents per gram of extract (mg AAE/g) (Prieto et al., 1999).

### 2.11 Antimitotic activity

#### 2.11.1 Anti-germination power evaluation test

The evaluation of the antimitotic activity of *M. vulgare* extract was done against *Lepidium sativum* seeds by preparing different concentrations of the extracts by dilution with distilled water. Initially, a 10 mg/ml concentration solution was prepared by diluting the extracts in 4 ml of distilled water. Subsequently, four dilutions were prepared with concentrations of 1,000, 100, 10, and 1 μg/mL. Control solutions including distilled water, colchicine (2.5 mg/ml), and methotrexate (5 mg/ml) were also prepared. Subsequently, these solutions were utilized to soak the watercress seeds for the assessment of antimitotic activity. After 72 h, the antimitotic activity of the extracts was determined by treating the *Lepidium sativum* seeds in Petri dishes with the respective plant decoction suspensions after which the length of the germinated Lepidium sativum rootlets was then measured using sterile forceps (Kumar and Singhalb, n.d.).

#### 2.11.2 Determination of the inhibition rate

The activity of the extract is assessed through the quantification of the percentage inhibition of cell growth. Noteworthy, this quantification involved comparing the growth of cells treated with the extract to that of a control batch by employing the formula below:

$$\%\;inhibition=\frac{LC-LT}{LC}*100$$

Where:

**LC:** length of control rootlets

**LT:** length of treated rootlets

#### 2.11.3 Determination of mitosis index

The mitosis index was determined by comparing the size of shoots from seeds soaked in the extract with those soaked in water (negative control). The measurements were conducted after 144 h of incubation in a dark and light-free environment, ensuring optimal conditions for observation and analysis (Mbayo et al., 2016). The value of the mitosis index was then determined using the formula below:

$$Im=\frac{S_{extract}\left(mm\right)}{S_{water}\left(mm\right)}$$

where:

**S**_**extract**_ **(mm):** Size of soaked seeds soaked in the extract;

**S**_**water**_ **(mm):** Size of soaked seeds soaked in water.

### 2.12 Determination MIC, MBC, and MFC

The Minimum Inhibitory Concentration (MIC) of the *M. vulgare* extract was determined using the microdilution method in a 96-well microplates, in accordance with the protocol utilized in the study by Kotan et al. (Kotan et al., 2008). The MIC represents the lowest concentration of the plant extract which visibly inhibit the growth of the microorganism being evaluated. To determine the MIC, standard solutions of the extracts were prepared by dilution in 10% dimethyl sulfoxide (DMSO) to obtain concentrations of extracting ranging from 75 to 2.35 mg/ml. For the evaluation of the bacteria susceptibility, the dilutions were incorporated into Mueller-Hinton basal medium, while for fungal susceptibility testing, the Sabouraud bouillon medium was utilized. Each well of the microplate was filled with 100 µL of the respective dilution, and subsequently, 100 µL of the inoculum containing a microbial count of 10^6^ colony-forming units per milliliter (CFU/ml) was sequentially introduced into each well. The microplate was subjected to a 24-h incubation period at 37°C after which 10 µL of resazurin, a colorimetric indicator of bacterial growth, was introduced into each well. Subsequently, the microplates were further incubated for 2 hours at 37°C, after which color change from deep violet to pink, was monitored as it indicates the absence of microbial growth, signifying the MIC. Noteworthy, the experimental setup included growth and sterility control wells and the MIC determination process was performed twice for the *M. vulgare* extract. The determination of the MBC/MFC was performed by aseptically transferring 10 µL of the contents from wells without visible growth onto Mueller Hinton agar (MHA) and Sabouraud agar plates for bacteria and fungi respectively. Subsequently, the plates were incubated for 24 h at 37°C and 30°C for bacteria and fungi respectively, and the MBC and MFC were defined as the lowest concentration of the extract that resulted in a 99.9% reduction in CFU/ml compared to the control. Noteworthy, 250 mg of Terbinafine was utilized as the standard antifungal by dissolving in 10% DMSO. The evaluation of the antimicrobial efficacy of the varying concentrations of the extract was done by calculating the MBC/MIC or MFC/MIC ratio, where a ratio of less than 4 indicated a bactericidal/fungicidal effect, a ratio greater than 4 indicated a bacteriostatic/fungistatic effect, and a ratio equal to 4 suggested an indeterminate effect (Bissel et al., 2014).

### 2.12 Animals

The animal model employed in this study consisted of *Wistar rats* weighing between 195–260 g and *Albino mice* weighing between 20–30 g. The animals were housed in a dedicated animal facility located within a biological laboratory. The housing conditions provided a controlled environment with a photoperiod of 12 h of light followed by 12 h of darkness, and a temperature maintained at 22°C. The rodents were kept under standard breeding conditions, with unrestricted access to food and water.

The procedures used to perform this study agree with the international guidelines used for the use of laboratory animals. The ethical committee of the Faculty of Sciences of Meknes, Morocco, revised and approved this work under the ethical clearance 04/2019/LBEAS.

### 2.13 Antidiabetic activity

#### 2.13.1 Acute toxicity

The dosage of *M. vulgare* extract which guarantees efficacy without toxicity in the short term evaluated in normal mice. The experimental design involved the use of albino mice weighing between 20–35 g, which were subjected to a 14-h fasting period before the experiment. The mice were randomly divided into five groups, with each group consisting of six mice (three males and three females). The mice were subjected to treatment based on their groups as follows:

• **Group 1:** This group served as the control group, and they were orally administered a dose of pure water at 10 mL/kg.
• **Groups 2, 3,** and **4:** Those groups were orally administered *M. vulgare* extract at concentrations of 0.5 g/kg, 1 g/kg, and 2 g/kg, respectively.

Noteworthy, before the administration of the extract, the mice were weighed to ensure accurate dosage. Following the administration of the extract, the mice were meticulously monitored for 10 h to observe any signs of toxicity and were assessed regularly for any adverse effects or symptoms of poisoning. Subsequently, daily observations were carried out over 14 days to detect any delayed toxicity or long-term effects. The animals were carefully handled throughout this period and the experiment was conducted in strict compliance with the guidelines of the Organisation for Economic Co-operation and Development (OECD) (Tchoumtchoua et al., 2014).

#### 2.13.2 Antihyperglycemic effect

The antihyperglycemic effect of the plant was evaluated *in vivo* by utilizing the oral glucose tolerance assay as described by Bouhrim *et al.* (Bouhrim et al., 2021). Normal rats were randomly distributed into three groups, with each group consisting of six rats (three males and three females). The groups were designated as follows:

**Control group:** Rats in this group were administered a dose of filtered water at 10 mL/kg.

**Test group:** Rats in this group were orally administered the plant extract at a dosage of 0.8 **mL/kg.**

**Treated groups:** Rats in this group were orally administered either the decocted extract at a concentration of 400 mg/mL or glibenclamide at a concentration of 2 mg/mL.

The evaluation of the oral glucose tolerance was conducted as follows: the measurement of the baseline glycemia was done at time zero, the sequel to the administration of their respective regimens (filtered water, *M. vulgare*, or glibenclamide). Subsequently, another blood glucose measurement was taken after 30 min, following the administration of an overload of D-glucose (2 mg/kg). Blood glucose levels were further monitored at 60, 90, and 150 min post-glucose overload. The oral glucose tolerance test was conducted in the following manner: Initially, baseline glycemia was measured at t_0_, immediately after the administration of the respective treatment (filtered water, plant extract, or glibenclamide). Subsequently, another blood glucose measurement was taken after 30 min, following the administration of an overload of D-glucose (2 mg/kg). Blood glucose levels were further monitored at 60, 90, and 150 min post-glucose overload. The oral glucose tolerance test was performed as follows: glycemia was measured at t_0_, immediately after treatment of the tested product (filtered water, plant extracts or glibenclamide). Another blood glucose measurement was taken 30 min later, just after the animals were overloaded with D-glucose (2 mg/kg). Blood glucose levels were then monitored at 60, 90, and 150 min.

#### 2.13.3 Pancreatic α-amylase inhibitory activity

##### 2.13.3.1 *In vitro* test

The effect of the M. vulgare extract on the enzymatic activity of pancreatic α-amylase was investigated following the method described by Bouhrim *et al.* (Bouhrim et al., 2021). To perform the analysis, 200 µL of the plant extract solution at various concentrations (0.89, 0.45, 0.22, 0.11, and 0.06 mg/mL) or the acarbose solution at different concentrations (1, 0.8, 0.6, 0.4, and 0.2 mg/mL) was combined with 200 µL of a phosphate buffer solution (0.02 M, pH = 6.9). Subsequently, 13 IU of pancreatic α-amylase enzyme solution was added to all tubes, excluding a tube, which contained only the phosphate buffer instead of the enzyme solution. The tubes were then pre-incubated at 37°C for 10 min. Following the pre-incubation, a 200 µL volume of starch solution was introduced to each tube, and the mixture was further incubated at 37°C for 15 min. To halt the enzymatic reactions, 600 µL of DNSA (2.5%) was added to the tubes. The tubes were then immersed in a scalding water bath for 8 min. To terminate the reaction, a heat shock was applied by placing the tubes in an ice bath, followed by the addition of 1 mL of filtered water to each tube. A spectrophotometer was employed to determine the absorption spectrum at 540 nm in comparison to a blank that contained the buffer solution rather than the enzyme solution. The percentage inhibition of each extract and acarbose was assessed using the equation below:

$$Percentage\;of\;inhibitory\;activity=\left(\left({Ab}_{Control}-{Ab}_{Test}\right)\;/\;\left({Ab}_{Control}\right)\right)\times 100$$

Where;

**Ab**_**Control:**_ Absorption of enzyme activity in the absence of an inhibitor;

**Ab**_**Test:**_ Enzymatic activity absorption with the plant extract or acarbose.

##### 2.13.3.2 *In vivo* test

The inhibitory effect of *M. vulgare* decoction on pancreatic α-amylase was evaluated *in vivo* in healthy rats by examining its impact on the activity of the intestinal lumen. Healthy rats weighing between 180–250 g were subjected to a 14-h fasting period and were divided into three groups, with a total of six rats in each group, and an equal distribution of males and females (♂/♀ = 1):

The control group was orally administered filtered water at a dose of 10 mL/kg, while the medicated groups were orally administered the decocted extract of *M. vulgare* at a dose of 400 mg/kg, or acarbose at a dose of 10 mg/kg. Assessment of the oral starch tolerance was carried out as follows: The test substance (distilled water, aqueous extract, or acarbose) was administered to the rats at time t = 0 min. After a 30-min interval, a blood glucose measurement was taken immediately following the administration of a starch overload (3 g/kg) to the rodents. The subsequent changes in blood glucose levels were monitored at 60, 90, and 120 min (Daoudi et al., 2020).

### 2.14 Molecular docking methodology

An *in silico* evaluation of the compounds derived from the *M. vulgare* decoction was done against antimitotic, antidiabetic, and antimicrobial targets. Following the identification of the targets, their respective structures were retrieved from the RCSB protein data bank using the PDB IDs 1XO2, 4W93, and 1AJ6. The preparation of the protein before molecular docking was done using BIOVIA’s Discovery Studio Visualizer (Accelrys Software Inc, 2005) and Autodock tools (Morris et al., 2009). Notably, the preparatory steps included the removal of heteroatoms, cognate ligands, and water molecules, and subsequent optimization of the structure. Similarly, the structure of the ligands was drawn in ChemDraw Ultra (CambridgeSoft, 2009a), and was subjected to preparatory steps including energy minimization using Chem3D Pro (CambridgeSoft, 2009b). Subsequently, the ligands were converted to pdbqt files (Zentgraf et al., 2007) using OpenBabel (Morris et al., 2009; Ferreira et al., 2015), while the molecular docking was performed using AutoDock Vina (Yusuf et al., 2008; Trott and Olson, 2010; Eberhardt et al., 2021). Following the formation of the complexes after docking, the interactions between the ligands and the protein were visualized and analyzed using BIOVIA’s Discovery Studio Visualizer.

### 2.15 Statistical analysis

The data presented in this study are expressed as means ± standard error of measurement (SEM). All statistical analysis was performed using one-way analysis of variance (ANOVA) followed by Tukey’s *post hoc* test. Noteworthy, significance levels were determined with *p* < 0.05, *p* < 0.01, and *p* < 0.001 considered as statistically significant.

## 3 Results and discussion

### 3.1 Quality control assessment of *M. vulgare* leaves

The quality control assessments conducted on the retrieved leaves of *M. vulgare* included the determination of its moisture content (TH), pH, acidity, ash, and ICP-AVE. The results of this assessment are presented in Tables 2, 3. As evident in Table 2, the TH of *M. vulgare* leaves was found to be 22.56 ± 0.25%, indicating a high water content in the plant material. The pH of the *M. vulgare* extract was slightly acidic, with a value of approximately 5.945 ± 0.007, however, the pH value was in compliance with the quality standards of AFNOR (AFNOR, 1977; Kakoma et al., 2014). Determination of the titratable acidity revealed values of 0.1033 ± 0.9212, which was used to evaluate the characteristics, quality, absorption capacity, and solubility of many substances (Kroeze, 2020). Similarly, the percentage of ash in *M. vulgare* extract which provides information about the mineral content that remains after organic matter is volatilized at high temperatures was found to be 22.562 ± 0.25%. As presented in Table 3, analysis of the heavy metal content of the extract revealed Fe possessed the highest content with a value of 0.5498 mg/g, while Cu was the second-highest with a value of 0.0087 mg/g. Other heavy metals analyzed are presented in Table 3, interestingly, they were all found to be within the permitted range of FAO/WHO regulatory standards. Hence, rendering the extract suitable for direct consumption, as an ingredient in food processing, or for repackaging if necessary.

**TABLE 2:** Quality control assessment of *M. vulgare* (TH, pH, Acidity, Ash, and MO).

Scientific name	TH (%)	pH	Acidity	Ashes MM (%)	MO (%)
*M. vulgare*	22.562 ± 0.25	5.945 ± 0.007	0.1033 ± 0.9212	73.6 ± 0.032	26.4 ± 0.0231

TH%, humidity level; Ashes MM (%), ash content; MO (%), organic matter content.

**TABLE 3:** Heavy metal content (ICP) and FAO/WHO maximum limit (2009).

Heavy metals	Heavy metal content (mg/g)							
	Arsenic (As)	Chromium (Cr)	Antimony (Sb)	Lead (Pb)	Cadmium (Cd)	Iron (Fe)	Copper (Cu)	Titanium (Ti)
*M. vulgare*	0.0062	0.0018	0.0023	Undetectable	Undetectable	0.5498	0.0087	0.002
Maximum limit	1	0.3	2	20	3	1	-	

### 3.2 Phytochemical screening

The qualitative screening of phytochemicals enables the identification of the classes of compounds present in an extract. To this end, qualitative reactions were employed to determine the phytochemicals present in the extract of *M. vulgare,* and the results are presented in Table 4. The results revealed the presence of primary metabolites including polysaccharides, reducing sugars (glucose and fructose), proteins, and lipids in varying concentrations. Similarly, the presence of secondary metabolites including flavonoids, tannins, mucilages, sterols and triterpenes, coumarins, and saponins were confirmed, however, alkaloids were found to be absent (Table 4). Interestingly, the results of this study are consistent with those of Akther *et al.* and Fayyad *et al.* who reported in their study that *M. vulgare* is rich in gallic tannins, catechin tannins, sterols, and triterpenes and flavonoids (Akther et al., 2013; Fayyad et al., 2014). Tannins and flavonoids are widely explored for their pharmacological properties including antioxidant, antiviral, antitumor, anti-inflammatory, anti-allergic and anti-cancer activities (Khanbabaee and van Ree, 2001; Crozier et al., 2009). Furthermore, numerous *in vitro* and *in vivo* studies show that polyphenols can modulate carbohydrate metabolism and have antidiabetic properties (Hanhineva et al., 2010; Martel et al., 2017).

**TABLE 4:** Phytochemical screening of the *M. vulgare* extract.

Families			*M. vulgare* L
Primary Metabolites	Polysaccharide		++ (Starch)
	Reducing sugar (glucose, fructose)		+++
	Protein	Reaction of buiret	++
		Xanthoprotein reaction	++
	Lipids Lieberman Burchard reaction		+
Secondary Metabolites	Tannins		++
	Tannins Catechism		++
	Leucoanthocyanins		++
	Mucilage		+
	Cyanidia reaction		Flavanols
	Reducing Compounds		++
	Saponosides		Still
	Alkaloids		—
	Gallic tannins		++
	Bones and holosides		++
	Flavonoids		+++
	Coumarins		—
	Sterols and triterpenes		+

+++, Very abundant; ++, abundant; +, low; —, absent.

### 3.3 Total polyphenols, flavonoids, and tannins contents of *M. vulgare*

Quantitative analysis of the classes of phytochemicals present in the aqueous extract of *M. vulgare* was conducted and the results are presented in Table 5. Notably, the yield of the aqueous extract was found to be significant, with a value of 17.673 ± 0.48. Classes of phytochemicals present in the extract include a high content of polyphenols (488.432 ± 7.825 mg/EAG/g), flavonoids (25.5326 ± 1.317 mg/EQ/g), and tannins (23.966 ± 0.187 mg/EV/g). The results of the quantitative analysis were found to be consistent with that of the qualitative analysis, with the values also being much higher than that reported by Matkowski *et al.* and Wojdylo *et al.* whose study revealed that the methanolic extracts of *M. vulgare* had polyphenolic contents of 63.4 ± 1.7 mg/EAG/g and 3.86 ± 0.05 mg/EAG/g respectively (Wojdyło et al., 2007; Matkowski et al., 2008). Conversely, the flavonoid content of the extract was found to be lower than that reported in the study of Elberry *et al.* in which they reported a value of 15.53 ± 0.67 mg/EAG/g study (Elberry et al., 2015). Variations in the values could be due to many intrinsic and extrinsic factors like genetic and environmental factors, and this could influence the quality and quantity of chemical composition of phenolic compounds in plant material. Other factors related to harvesting, drying, and processing may also be responsible (Ouedraogo et al., 2021).

**TABLE 5:** Polyphenolic, flavonoid and tannin content of *M. vulgare* aqueous extract.

Extract yield (%)	Polyphenol (mg/EAG/g)	Flavonoid (mg/EAG/g)	Tannin (mg/EAG/g)
**17.673 ± 0.48**	488.432 ± 7.825	25.5326 ± 1.317	23.966 ± 0.187

The percent ratio of actual yield to the theoretical yield.

### 3.4 Chromatography of aqueous extract of *M. vulgare* HPLC/UV-ESI-MS

The identification of the phenolic composition of the aqueous of *M. vulgare* leaves was conducted using HPLC/UV-ESI-MS analysis. The resulting chromatographic profile, which reveals the presence of 19 chemical compounds presented in Table 6, is depicted in Supplementary Figure S1 (supplementary provided). Notably, the major phyto-compounds present in the extract were catechin (12.03%), maleic acid (11.18%), luteolin (10.55%), apigenin (10.55%), salicylic acid (8.01%), biotin (7.24%), caffeic acid (6.36%), and vanillic acid (5.03%), these eight compounds accounted for a significant proportion, totaling 70.95% of the total identified compounds (96.78%). Interestingly, a study by Benzidane et al., 2020 on the methanolic extract of *M. vulgare* leaves collected in Algeria revealed the presence of ferulic acid (Nadia Benzidane, Ridha Smahi, Boudjemaa Zabouche, Abdelhalim Makrouf, 2020), however, the compounds were found to be present in the current study with a very low percentage (0.77%). They also reported the presence of catechin in the hexane extract, which is in tandem with the compounds identified in our aqueous extract. A recent study in Saudi Arabia reported that the methanolic extract of the leaves of *M. vulgare* contains luteolin-7-O-D-glucoside as the third major compound, however, luteolin was found to be present in this and was not linked to any sugar moiety. Furthermore, a study by Rezgui *et al.* in Tunisia corroborated the presence of several phenolic molecules in *M. vulgare* leaves, including apigenin, ferulic acid, coumaric acid, caffeic acid, and luteolin (Rezgui et al., 2020), which are in line with the compounds identified in our study. These compounds have been extensively investigated for their pharmacological properties, such as anticancer, anti-inflammatory, antibacterial, antiviral, and antiseptic activities, as documented in previous scientific studies (Karryev et al., 1976; Pukalskas et al., 2012; Yousefi et al., 2013). Noteworthy, the observed variation in the reported compared to that of other studies could be attributed to intrinsic and/or extrinsic factors.

**TABLE 6:** Chemical composition of *M. vulgare* aqueous extract identified by HPLC/UV-ESI-MS.

N^0^	RT	M/Z	[M-1]^+^	M	[M+1]^+^	Compounds	AR%
1	3,99	209	207	208	209	Chalcone	0,86
2	4,24	151	149	150	151	Ribose	1,88
3	4,82	159	159	160	161	Pimelic acid	4,17
4	5,55	139	137	138	139	Salicylic acid	**8,01**
5	6,22	243	243	244	245	Biotin	**7,24**
6	6,75	117	115	116	167	Maleic acid	**11,18**
7	9,74	139	137	138	139	Urocanic acid	1,33
8	10,51	295	293	294	295	Embeline	3,6
9	11,42	191	191	192	193	Quinic acid	0,64
10	15,1	189	189	290	291	Catechin	**12,03**
11	16,48	179	179	180	181	Caffeic acid	**6,36**
12	17,35	167	167	168	169	Vanillic acid	**5,03**
13	17,84	197	197	198	199	Syringic acid	4,17
14	19,83	165	163	164	165	Coumaric acid	4,68
15	20,11	195	193	194	195	Ferulic acid	0,77
16	23,47	319	317	318	319	Myricetin	0,72
17	25,11	387	387	388	389	Luteolin	**10,55**
18	25,32	287	285	286	287	Kaempferol	3,01
19	25,46	269	269	270	271	Apigenin	**10,55**
Total (%)							96,78

Maximum Concentration Area of compound in percentage.

### 3.5 Antioxidant activity of *M. vulgare*

The assessment of the antioxidant activity of plant extract was done using three different methods namely DPPH, FRAP, and TAC tests, the results of which are presented in Table 7. The results revealed a positive correlation between the inhibitory effect of the free radical DPPH and the quantity of the M. vulgare extract. The antioxidant activities of the extract and ascorbic acid were quantified by the determination of their IC_50_, which represents the concentration of the extract required to reduce 50% of the DPPH free radical. Noteworthy, a lower IC_50_ value indicates a higher antioxidant effect. Interestingly, the results of this study revealed that the decoction of *M. vulgare* exhibited significant antioxidant power (IC50 = 1.1815 ± 0.621 mg/mL), although lower than that of the ascorbic acid (IC50 = 0.323 ± 0.411 mg/mL). The results of this study differ slightly from those reported in a study by Boudjelal *et al.* in which an IC50 value of 0.49 ± 0.517 mg/mL was reported for the aerial part of the methanolic extract of *M. vulgare* harvested in M'Sila, southern Algeria (Boudjelal et al., 2013). Evaluation of the reducing power via measuring its ability to transfer an electron or a hydrogen atom, thereby reducing Fe (III) to Fe (II) in the presence of the K_3_Fe(CN)_6_ complex, was done and the results are presented in Figure 3B. Notably, a remarkable increase in the reducing power of *M. vulgare* was noticed in concomitant with the increasing concentrations of the extract. The ascorbic acid and the decoction exhibited effective concentrations with respective values of EC50 = (0.15 ± 0.24) mg/mL and EC50 = (1.5 ± 0.203) mg/mL. An earlier study on *M. vulgare* extract reported that it possessed significant antioxidant activity, with an EC_50_ of 0.51 ± 0.24 mg/mL (Ghedadba et al., 2014), this activity was attributed to the phenylpropanoid glycosides, which are reputed for their potent antioxidant activities. The TAC of *M. vulgare* extract was determined by the phosphomolybdate test which is based on the ability of the extract to reduce molybdenum Mo (VI) present as molybdate ions MoO_4_^2−^, to molybdenum Mo(V) MoO_4_^2+^, and the subsequent formation of a green complex [phosphate Mo(V)] at an acidic pH. The results of this study revealed that the decoction shows a TAC of 50.550 ± 3.746 mg EAA/gE, hence, indicative of the remarkable antioxidant capacity of the aqueous extract. However, the results of this study were not in tandem with that reported in a study by Amessis-Ouchemoukh *et al.*, obtained from studying the TAC of the methanolic and acetone extracts of the aerial part of *M. vulgare* which showed high TAC with 101.82 ± 1.75 and 85.71 ± 1.35 (Amessis-Ouchemoukh et al., 2014). The antioxidant activities demonstrated by the aqueous extract of M. vulgare, as assessed through DPPH, FRAP, and TAC tests, hold potential applications in the food and pharmacological industries. These findings contribute to the understanding of the antioxidant properties of *M. vulgare* and its potential as a natural source of antioxidants.

**TABLE 7:** TAC of the aqueous extract of *M. vulgare*.

	DPPH IC_50_ (mg/mL)	FRAP EC_50_ (mg/mL)	CAT (mg/EAG/gE)
Plant extract	1.18 ± 0.621	1.5 ± 0.203	50.550 ± 3.746
Ascorbic acid	0.32 ± 0.411	1.15 ± 0.24	

### 3.6 Antimitotic activity of *M. vulgare*

The antimitotic effect of *M. vulgare* extract was carried out by the *Lepidium sativum* method which was proposed in 1972 by GAGIU for a preliminary selection of molecules acting on plant growth without presuming their precise place of action. Several authors have contributed to the study of the validity of this test, and it is widely used as it is relatively simple and fast. Additionally, the seed of *Lepidium sativum* has only one rootlet, the appreciable growth of which allows easy measurement. This test is conducted by measuring the length of the root of a germinated *Lepidium* seed placed in a medium containing the molecules to be tested. The monitored percentage of growth inhibition is estimated by comparison with a control. Colchicine, with an already-known antimitotic effect, was used as a positive control. The antimitotic activity of the *M. vulgare* decoction was evaluated by determining the inhibition index. As evident in Table 8, an increase in the concentration of the extract was concomitant with an increase in the percentage inhibition, and the extract was found to possess better antimitotic activity compared to colchicine with 90.184 ± 0.164% and 95.37 ± 0.19% at 10 mg/mL and 1.5 mg/mL respectively. Conversely, the IC_50_ values obtained for the decocted extract were higher for colchicine compared to the decoction with the values 1.8 ± 0.11 mg/mL and 3.688 ± 0.12 mg/mL respectively. The results of the mitotic index of the *M. vulgare* extract are presented in Table 9, with the extract exhibiting a higher value compared to the colchicine but lower compared to the negative control (distilled water). It is worth noting that a smaller mitotic index indicates a higher activity. Overall, the results of this study render the extract of *M. vulgare* fit for exploration as an antimitotic.

**TABLE 8:** Percentage of mitotic indices of *M. vulgare* and control extracts (distilled water).

Extracts	Mitotic indices (%)				
Colchicine (T+)	6,74%				
Distilled water (T-)	95%				
	**C = 0,001 mg/mL**	**C = 0,01 mg/mL**	**C = 0,1 mg/mL**	**C = 1 mg/mL**	**C = 10 mg/mL**
*M. vulgare* L	90,8%	90,54%	74,63%	53%	30,19%

C = Control values used.

**TABLE 9:** Percentage of inhibition of *M. vulgare* extract.

Plant	Inhibition percentage (%)				
*M. vulgare* L	**C = 0.001 mg/mL**	**C = 0.01 mg/mL**	**C = 0.1 mg/mL**	**C = 1 mg/mL**	**C = 10 mg/mL**
	9.202 ± 0.114	17.791 ± 0.130	38.650 ± 0.212	67.484 ± 0.089	90.184 ± 0.164

C = Control values used.

### 3.7 Antimicrobial activity of *M. vulgare*

#### 3.7.1 Antibacterial activity

Evaluation of the MIC was conducted using the microdilution method, and the results are presented in Table 10. Notably, all the strains examined exhibited varying degrees of sensitivity to the aqueous extract of *M. vulgare*, as evident by the variability in the MIC values obtained. The classification of MICs was done based on the established criteria described in previous studies (Sartoratto et al., 2004; Duarte et al., 2007; de Oliveira Pedro et al., 2013; Wang et al., 2017). The antibacterial effect of *M. vulgare* aqueous extract was classified as high (MIC 75 μg/mL) against *Klebsiella pneumoniae*, *Escherichia coli*, *Pseudomonas aeruginosa*, and *Staphylococcus epidermidis*. A moderate antibacterial effect (MIC 37.5 μg/mL) was observed against *Streptococcus agalactiae*, while a low antibacterial effect (MIC 18.75 μg/mL) was observed against *Proteus mirabilis*. It is worth noting that the MIC values indicate the lowest concentration of the extract that inhibited the visible growth of the bacterial strains. Furthermore, the MBC values were found to be less than 75 μg/mL for all tested bacterial strains.

**TABLE 10:** MIC and MBC values (µg/ml) of the *M. vulgare* extract.

Bacterial strains	References	MIC	MBC
*Klebsiella pneumoniae*	3DT1823	75	<75
*Streptococcus agalactiae* (B)	7DT1887	37,5	<75
*Proteus mirabilis*	2DS5461	18,75	<75
*Escherichia coli sauvage*	3DT1938	75	<75
*Pseudomonas aeruginosa*	2DT2138	75	<75
*Staphylococcus epidermidis*	5,994	75	<75

#### 3.7.2 Antifungal activity

The antifungal impacts of *M. vulgare* aqueous extract were also investigated on certain fungal strains and the results are presented in Table 11. The results of the study revealed the extract exhibited significant antifungal activity and MFC of 37.5 μg/mL against the *condidat strains*; *Candida tropicalis*, *Candida krusei*, *Candida albicans*, *Candida parapsilosis*, *Saccharomyces cerevisiae* and *Aspergillus niger*, except *Candida dubliniensis* which was inhibited by a higher concetration with a MIC value of 75 μg/mL. Notably, the lowest MFC value of 9.375 μg/mL was observed against *Candida parapsilosis*, *Saccharomyces cerevisiae*, *Aspergillus niger*, which is indicative of their strong antifungal activity. A moderate MFC value of 18.75 μg/mL was observed against *Candida krusei* while the highest MFC values were found to be less than 75 μg/mL against *Candida tropicalis*, *Candida dubliniensis*, and *Candida albicans*.

**TABLE 11:** MIC and MFC values (µg/mL) of the extract.

Fungal strains	References	MIC	MFC
*Candida tropicalis*	Ct	37.5	<75
*Candida dubliniensis*	Cd	75	<75
*Candida krusei*	Ckr	37.5	18.75
*Candida albicans*	Ca	37.5	<75
*Candida parapsilosis*	Cpa	37.5	9.375
*Saccharomyces cerevisiae*	Sacc	37.5	9.375
*Aspergillus niger*	AspN	37.5	9.375

This study demonstrated the antimicrobial properties of the aqueous extract of *M. vulgare* against the tested bacterial and fungal strains, this is attributable to the active constituents of this plant (Dib et al., 2021). Previous studies conducted on *M. vulgare* have identified catechin as the main component and it is present alongside a variety of bioactive substances including alkaloids, steroids, terpenes, and tannins (Meyre-Silva and Cechinel-Filho, 2010). Interestingly, the antibacterial activities of these compounds have been reported (Kurbatova et al., 2003; Djahra et al., 2013; Masoodi et al., 2015). Furthermore, previous studies have shown that *M. vulgare* extracts have antimicrobial activity against pathogenic bacterial and fungal strains (Molina-Salinas et al., 2006; Bouterfas et al., 2016; Al-Tohamy et al., 2018; Bouterfas et al., 2018).

### 3.8 Antidiabetic activity of *M. vulgare*

#### 3.8.1 Antihyperglycemic effect

**FIGURE 2:**
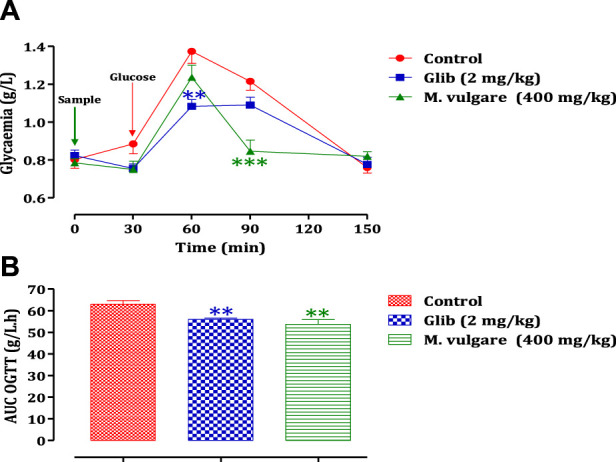
Change in postprandial glycemic **(A)** and the post-prandial curve area **(B)** in healthy rats after taking the products test (decocted and glibenclamide). The values are SEM averages. (*n* = 6). ***p < 0.001; **p < 0.01: when compared to the control.

The administration of *M. vulgare* aqueous extract (400 mg/kg) 30 min before glucose overload resulted in a significant reduction in postprandial hyperglycemia at 60 min (*p* < 0.01) and 90 min (*p* < 0.001). Similarly, glibenclamide, a reference antidiabetic drug, significantly inhibited postprandial hyperglycemia at 60 min (*p* < 0.01) and 90 min (*p* < 0.05) following glucose overload. By 150 min, as presented in Figure 2A, there were no significant differences in blood glucose levels among all rat groups compared to the normal rats at 60 min, 90 min, and 150 min. Furthermore, analysis of the area under the curves (AUC) showed a significant reduction (*p* < 0.01) in rats treated with *M. vulgare* aqueous extract compared to rats treated with distilled water. Similarly, the AUC of glibenclamide was significantly lower (*p* < 0.01) than that of animals treated with distilled water (Figure 2B). These findings suggest that the administration of *M. vulgare* extract effectively ameliorated elevated glucose levels in a manner similar to glibenclamide, indicating its potential antihyperglycemic activity. The observed decrease in elevated glucose levels is consistent with the results of a study in which they reported that ethanolic extracts of *M. vulgare* showed a promising antihyperglycemic effect. Noteworthy, the mechanisms that underpin the observed antihyperglycemic activity of *M. vulgare* are numerous and they include the modulation of insulin secretion by the beta cells of the islets of Langerhans as well as extrapancreatic and pancreatic effects of insulin. However, elucidating the precise mechanism is still an active area of research. HPLC analysis of *M. vulgare* extract revealed catechin, maleic acid, luteolin, apigenin, and salicylic acid as the main constituents. Interestingly, these bioactive compounds are likely responsible for the observed antihyperglycemic effect as they been reported possess antidiabetic properties and contribute to the regulation of glycemia (Olmedilla et al., 1997; Kumari et al., 2012). Quercetin, for instance, has been reported to exhibit insulin-like activity or increase insulin secretion (Khatware and Annapurna, 2014). Therefore, the synergistic effect of these bioactive compounds in *M. vulgare* extract likely contributes to its antihyperglycemic activity. In this regard, there is a clear antihyperglycemic effect of *M. vulgare* extract, which reinforces its traditional utilization in the control of diabetic patients.

#### 3.8.2 Pancreatic α-amylase inhibitory activity

##### 3.8.2.1 *In vitro* test

**FIGURE 3:**
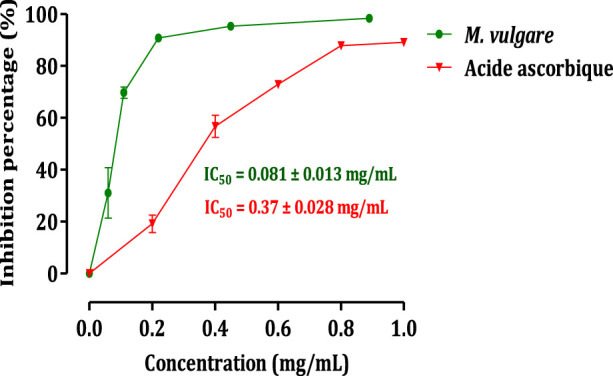
Inhibitory effect on the activity of α-amylase by *M. vulgare* extract and acarbose, *in vitro*. The values are averages ± SEM, (*n* = 3).

The impact of *M. vulgare* extract on the activity of pancreatic α-amylase is illustrated in Figure 3. The results demonstrate that the extract of *M. vulgare* significantly suppresses the activity of pancreatic α-amylase, with an IC50 value of 0.081 ± 0.013 mg/mL. This inhibitory effect on pancreatic α-amylase activity is even more pronounced compared to acarbose, a known α-amylase inhibitor, with an IC50 value of 0.37 ± 0.03 mg/mL. Pancreatic α-amylase plays a crucial role in the digestion of starch and glycogen. Inhibition of this enzyme’s activity involved in carbohydrate digestion can be an effective strategy for the management of carbohydrate assimilation disorders, such as diabetes mellitus (Ouassou et al., 2021). Therefore, the potent inhibitory activity of *M. vulgare* extract on pancreatic α-amylase, as demonstrated in this study, suggests its potential therapeutic use in the treatment of conditions related to abnormal carbohydrate metabolism. The observed activity of *M. vulgare* extract in inhibiting pancreatic α-amylase can be attributed to the presence of various bioactive substances within the extract. These bioactive compounds likely interfere with the formation of monosaccharides during carbohydrate digestion, leading to a reduction in blood glucose concentration and indicating a hypoglycemic activity. Further research is needed to identify and isolate the specific bioactive compounds responsible for the α-amylase inhibitory activity of *M. vulgare* extract and to explore their potential application in the management of carbohydrate-related disorders. Overall, the findings from this study highlight the potential of *M. vulgare* extract as a natural inhibitor of pancreatic α-amylase, offering prospects for the development of novel therapeutic approaches in the management of carbohydrate assimilation disorders such as diabetes mellitus.

##### 3.8.2.2 *In vivo* test

**FIGURE 4:**
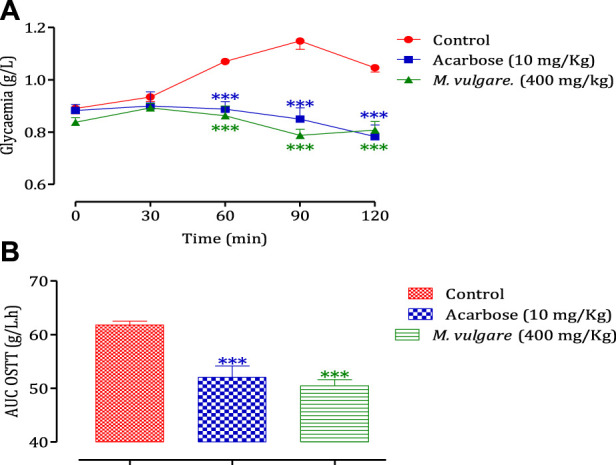
Effect of *M. vulgare* L and acarbose on postprandial blood glucose variation in normal rats **(A)**, with a representation as areas under the curves **(B)**. The values are SEM averages (*n* = 6) ***p < 0.001: compared to the control.

To investigate the influence of the intestinal environment on the inhibitory activity of *M. vulgare* on α-amylase, an *in vivo* test was conducted. The administration of *M. vulgare* extract (400 mg/kg), 30 min before starch overload in healthy rodents. The results demonstrated a significant reduction in postprandial hyperglycemia in the rodents treated with *M. vulgare* extract compared to the group pretreated with distilled water. The reduction in blood glucose levels was observed at 60, 90, and 150 min (*p* < 0.001 for all time points). Similarly, the administration of acarbose also led to a significant decrease in postprandial hyperglycemia at 60, 90, and 150 min compared to the rats treated with water (*p* < 0.001 for all time points) (Figure 4A). Moreover, the area under the curve was substantially less (*p* < 0.001) in rodents given *M. vulgare* than in rats treated with distilled water. The area under the curve for acarbose was markedly (*p* < 0.001) low compared to the area under the curve in animals treated with water (Figure 4B). The results of the inhibitory effect of *M. vulgare* extract on pancreatic α-amylase were confirmed *in vivo*, which is in accordance with prior *in vitro* experiments. Pancreatic α-amylase is one of the major enzymes in the human body and its inhibition is considered an important strategy in managing blood glucose levels. Therefore, it might be the best strategy to manage type 2 diabetes (Dwek et al., 2002). In fact, the *in vivo* hypoglycemic effect found for our extract could be related to the bioactive molecules responsible for this effect. These findings suggest that the presence of polyphenolic molecules may have a potentially important role in diabetes management via the inhibition of *α-*amylase enzyme activities. Additionally, Costamagna et al. (2016) suggested that the hydrolysis of phenolic molecules during digestion contributes to the accumulation of shorter phenolic groups, which could improve their pharmacological properties (Costamagna et al., 2016). In addition, several flavonol glycosides have also been reported to have potent α-amylase inhibition activities *in vivo* and *in vitro* models (Braca et al., 2003; He et al., 2007).

### 3.9 Molecular docking

The docking scores of the eight major components of *M. vulgare* extract, which represents their binding affinities for the antimitotic protein (1XO2), antidiabetic protein (4W93), and antimicrobial protein (1AJ6, 2023) are presented in Supplementary Table S2 (Supplementary provided). It is worth noting that a lower docking score corresponds to higher binding affinities. The results showed that the binding energies of the ligands vary depending on the protein target. For instance, Leotulin has the lowest binding energy with the antimitotic protein (1XO2) with a value of −9.3 kcal/mol, indicating a strong binding affinity, whereas Maleic Acid has the lowest binding energy with the antimicrobial protein (1AJ6) with a value of −4.3 kcal/mol, indicating a relatively weaker binding affinity. Interestingly, Leotulin consistently exhibits the lowest binding energy across all three protein targets, suggesting that it has a high potential to be an effective drug candidate for these protein targets. Conversely, Maleic Acid consistently shows the weakest binding affinity among the ligands. Apigenin and Vanillic Acid have relatively consistent binding energies across the three protein targets, whereas the other ligands have more varied binding energies depending on the protein target. For example, Catechin has significantly lower binding energy with the antidiabetic protein (4W93) than with the other two protein targets. Biotin shows moderate binding energies across all three protein targets, with a slightly stronger binding affinity to the antimitotic protein (1XO2) than to the other two protein targets. Salicylic Acid has a similar binding energy to the antidiabetic protein (4W93, 2023) and antimicrobial protein (1AJ6), whereas it has a slightly weaker binding affinity to the antimitotic protein (1XO2). Leotulin consistently exhibits the strongest binding affinity across all three protein targets, while Maleic Acid shows the weakest binding affinity. The other ligands have varying binding energies depending on the protein target, with some showing consistent binding affinities across all three protein targets. Major interactions with good results are shown in Table 12.

**TABLE 12:** Interaction of antimitotic protein target with the major components of *M. vulgare* extract ligands showing the 3D and 2D structural view. Full Table in Supplementary Table S4.

Ligands with 1XO2	3D amino acid interactions view	2D amino acid interactions view
Apigenin	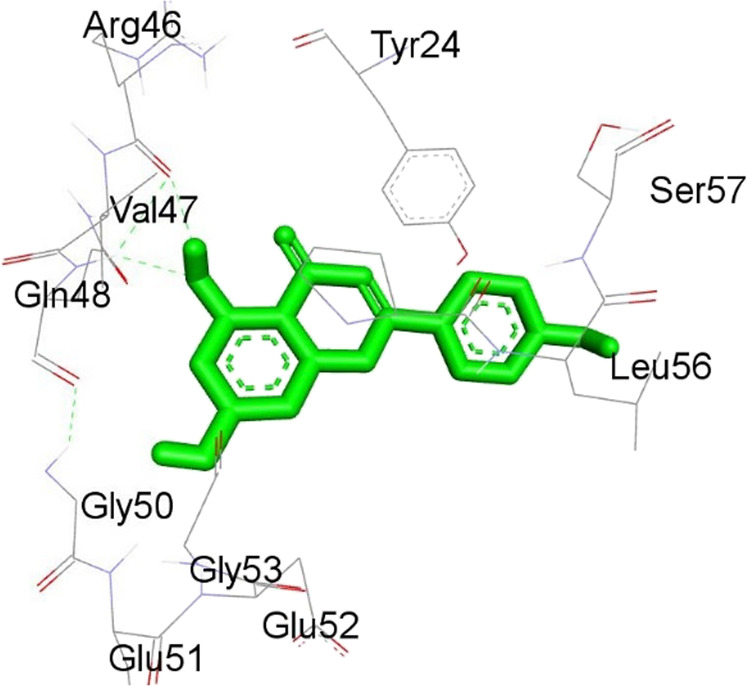	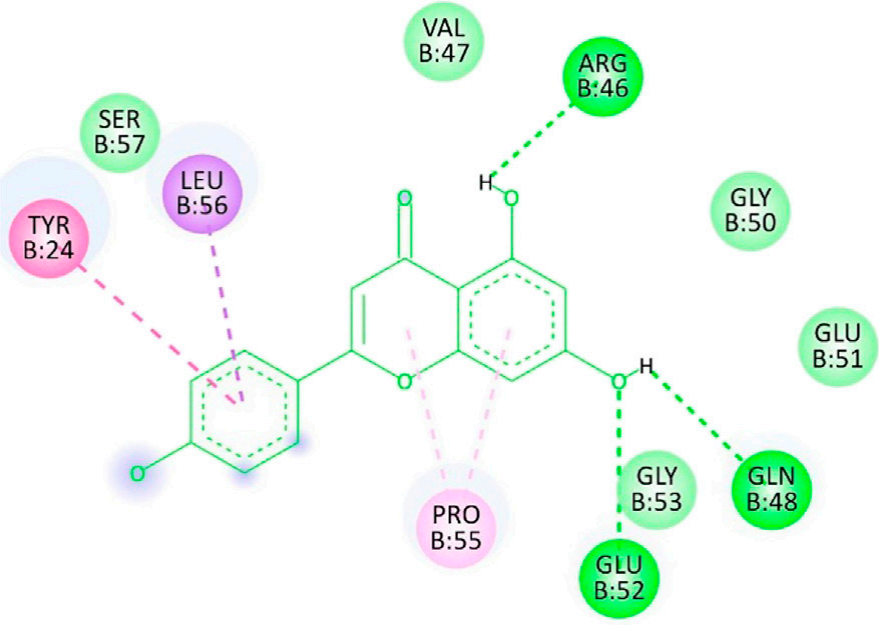
Leotulin	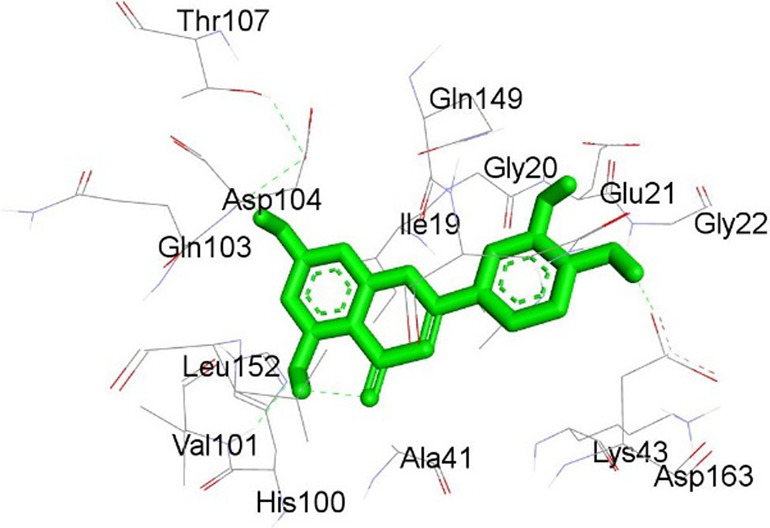	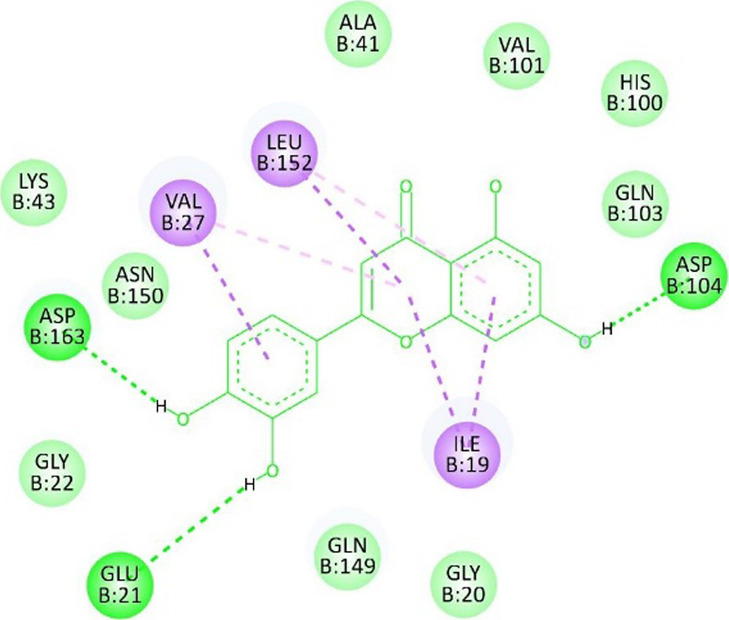

Supplementary Table S3 (Supplementary provided) shows the interaction of the antimitotic protein target (1XO2, 2023) with the major components of *M. vulgare* extract ligands, representing the binding pocket residue amino acids with distances and types of bonding interactions. Each ligand is listed with the amino acid residues that it interacts with in the binding pocket of the antimitotic protein, along with the distance between the ligand and the amino acid residues in Å. The types of bonding interactions are also listed, which can be either hydrogen bonding, π-bonding, alkyl bonding, or hydrophobic interactions. Apigenin interacts with several amino acid residues, including GLU52, ARG46, GLN48, LEU56, TYR24, and PRO55. The distances of these interactions range from 1.98 to 4.97 Å. The type of bonding interactions involved include H-bonds, π-bonds, and alkyl bonds. Biotin interacts with VAL27, LYS43, ALA162, and PHE98 amino acid residues. The distance between the ligand and these residues ranges from 4.19 to 5.44 Å. The type of bonding interactions involved are mainly alkyl bonds and hydrophobic interactions. Caffeic acid interacts with GLU61, VAL101, PHE98, VAL27, LYS43, and ALA162 amino acid residues. The distances between the ligand and these residues range from 2.56 to 5.24 Å. The type of bonding interactions involved includes H-bonds, alkyl bonds, and hydrophobic interactions. Catechin interacts with GLN48, ARG46, GLY53, and PRO55 amino acid residues. The distance between the ligand and these residues ranges from 2.16 to 5.33 Å. The type of bonding interactions involved includes H-bonds, alkyl bonds, and hydrophobic interactions. Leotulin interacts with several amino acid residues, including GLU21, ASP163, ASP104, ILE19, VAL27, LEU152, and others. The distances of these interactions range from 2.06 to 4.96 Å. The type of bonding interactions involved includes H-bonds, π-bonds, and alkyl bonds. Maleic acid interacts with ASP163, GLU99, and H-O amino acid residues. The distances of these interactions range from 2.10 to 2.87 Å. The type of bonding interactions involved includes H-bonds. Salicylic acid interacts with several amino acid residues, including LYS43, ASP163, GLU61, PHE98, VAL27, and others. The distances of these interactions range from 2.34 to 5.44 Å. The type of bonding interactions involved include H-bonds, π-bonds, hydrophobic bonds. Vanillic Acid showed interactions with ASP163 and GLU61 both have hydrogen bonds, while ALA162, VAL27 (both occurrences), and LEU152 have hydrophobic properties. Meanwhile, VAL77 has a π-bond, and VAL27 and LEU152 have alkyl bonds. All type of interactions is also shown in Supplementary Table S4 (Supplementary provided) as 3D and 2D. Major interactions with good results are shown in Table 13.

**TABLE 13:** Interaction of antidiabetic protein target with the major components of *M. vulgare* extract ligands showing 3D and 2D structural view. Full Table in Supplementary Table S5.

Ligands with 4W93	3D amino acid interactions view	2D amino acid interactions view
Apigenin	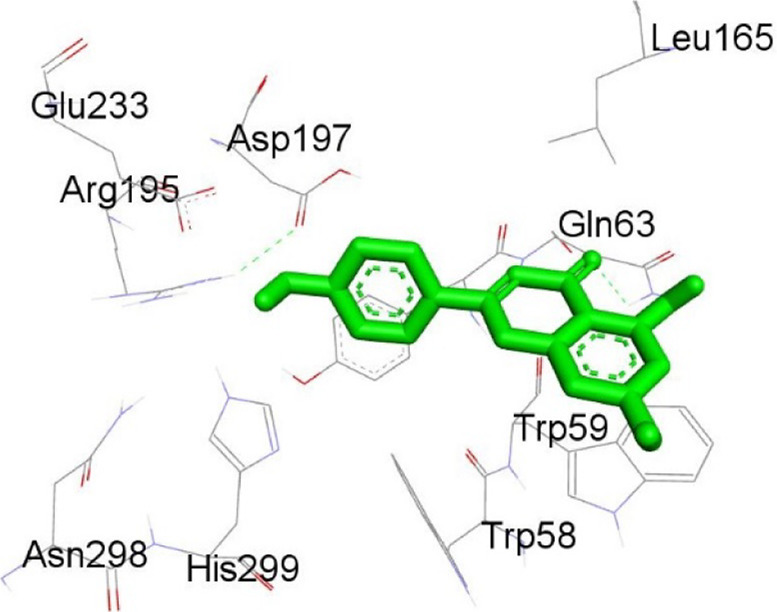	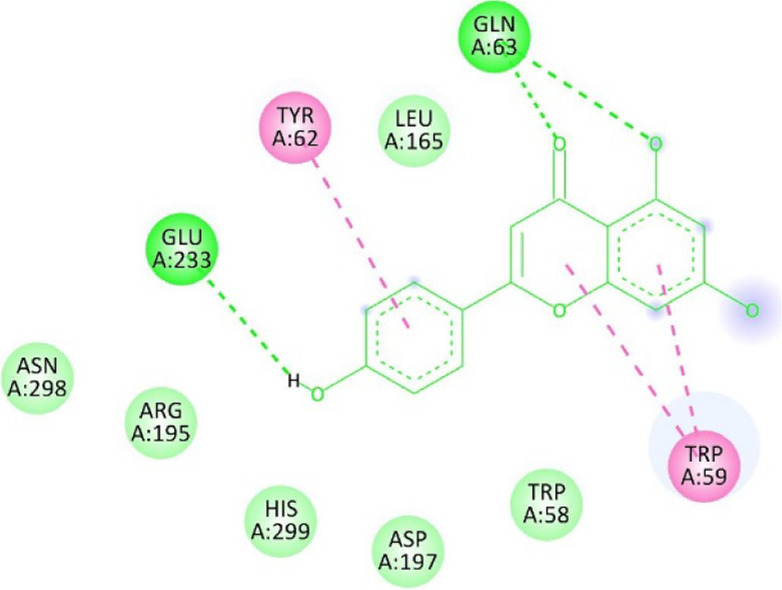
Leotulin	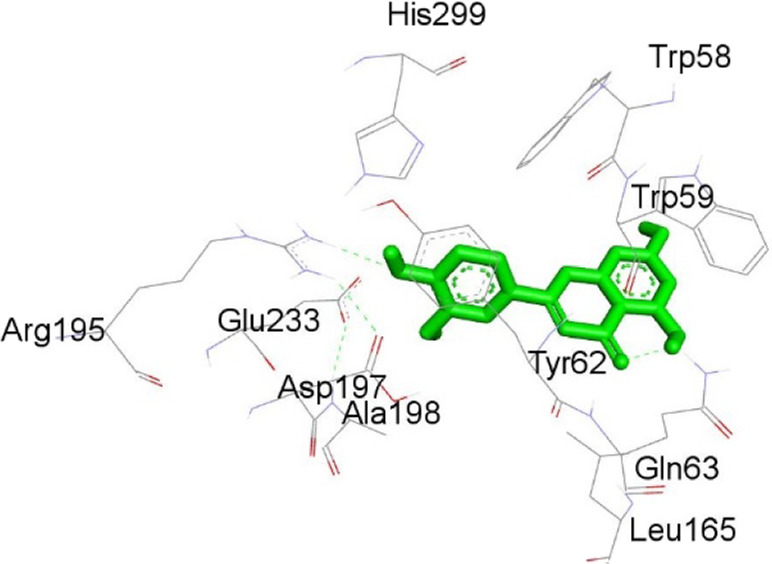	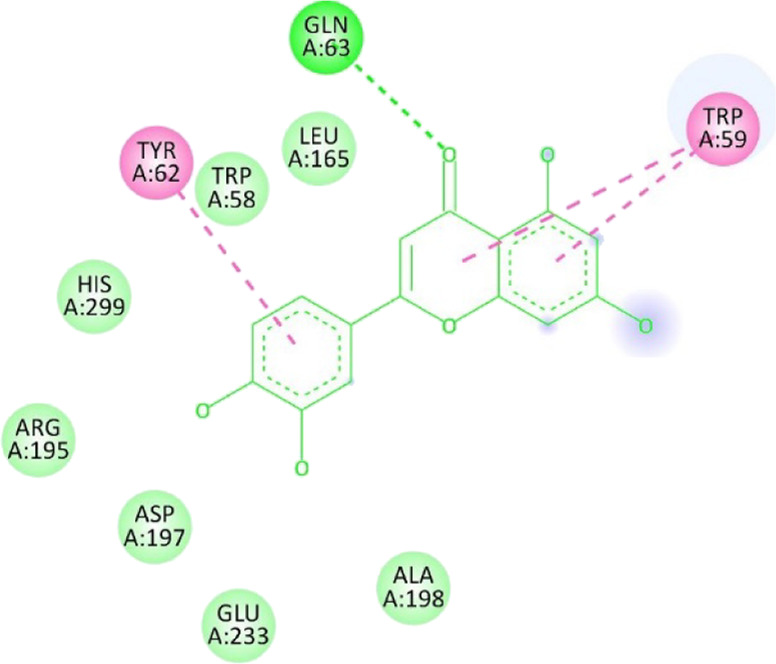

Supplementary Table S5 (Supplementary provided) shows the results of an experiment investigating the interaction between antidiabetic protein and the major components of *M. vulgare* extract ligands. The first ligand presented in the table is apigenin. The antidiabetic protein interacts with GLN63, GLU233, TRP59, and TYR62 amino acid residues of apigenin, and the distances between the amino acid residues and the ligand range from 2.47 to 5.23 Å. The bonding interactions between the protein and the apigenin ligand are mainly hydrogen bonds, except for a hydrophobic interaction and two types of alkyl bonds. The second ligand is biotin, and the antidiabetic protein interacts with ARG252, ARG421, PRO332, SER289, ASP402, and GLY334 amino acid residues of biotin. The distance between the ligand and the interacting amino acid residues ranges from 1.85 to 3.76 Å. The bonding interactions between the protein and biotin are mainly hydrogen bonds, except for two types of π-bonds. The third ligand is caffeic acid, and the antidiabetic protein interacts with ASP197 and TYR62 amino acid residues of caffeic acid. The distance between the ligand and the interacting amino acid residues ranges from 2.51 to 4.35 Å. The bonding interactions between the protein and caffeic acid are a hydrogen bond and an alkyl bond. The fourth ligand is catechin, and the antidiabetic protein interacts with ASP197, GLU233, TRP59, and TYR62 amino acid residues of catechin. The distance between the ligand and the interacting amino acid residues ranges from 2.05 to 4.66 Å. The bonding interactions between the protein and catechin are mainly hydrogen bonds, except for three types of alkyl bonds.

The fifth ligand is leotulin, and the antidiabetic protein interacts with GLN63, H-O, TRP59, and TYR62 amino acid residues of botulin. The distance between the ligand and the interacting amino acid residues ranges from 1.98 to 5.34 Å. The bonding interactions between the protein and leotulin are mainly hydrogen bonds, except for four types of alkyl bonds. The sixth ligand is maleic acid, and the antidiabetic protein interacts with ARG346, ASP317, and GLN302 amino acid residues of maleic acid. The distance between the ligand and the interacting amino acid residues ranges from 1.95 to 2.12 Å. The bonding interactions between the protein and maleic acid are all hydrogen bonds. The seventh and final ligand presented in the table is salicylic acid, and the antidiabetic protein interacts with ARG195, ASP197, GLU233, and TYR62 amino acid residues of salicylic acid. The distance between the ligand and the interacting amino acid residues ranges from 2.10 to 4.98. Vanillic Acid interacts with the ASP197 residue of the protein target through a hydrogen bond, with 2.32 Å. It also forms a second hydrogen bond with another ASP197 residue with 2.48 Å. Additionally; the ligand interacts with the GLU233 residue of the protein through a pi-bond, with 3.73 Å. Vanillic Acid binds to the TYR62 residue of the protein through an alkyl-bond, with 4.50 Å. Finally, the ligand interacts with the ALA198 and LEU162 residues of the protein through pi-bonds and alkyl-bonds, respectively, with distances of 3.75 and 4.98 Å. These interactions may play a role in the potential antidiabetic properties of the *M. vulgare* extract, as the ligand is able to interact with specific binding pocket residues of the protein target as shown in Supplementary Table 6S (Supplementary provided) as 3D and 2D representations. Major interactions with good results are shown in Table 14.

**TABLE 14:** Interaction of antimicrobial protein target with the major components of *M. vulgare* extract ligands showing 3D and 2D structural view. Full Table in Supplementary Table S7.

Ligands with 1AJ6	3D amino acid interactions view	2D amino acid interactions view
Apigenin	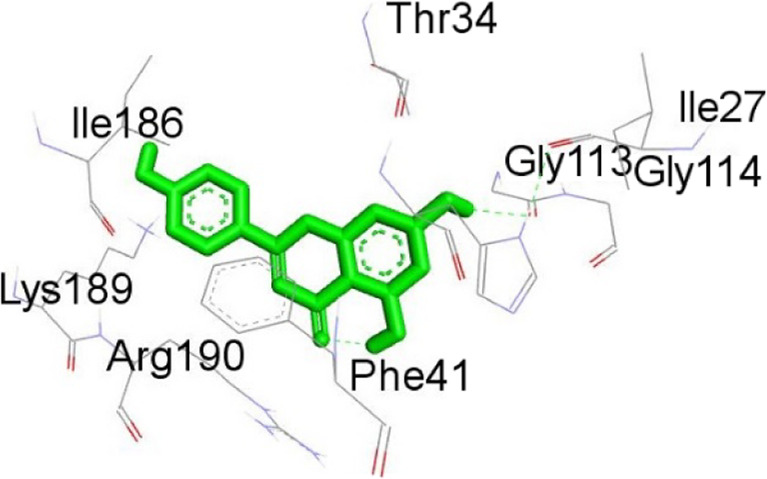	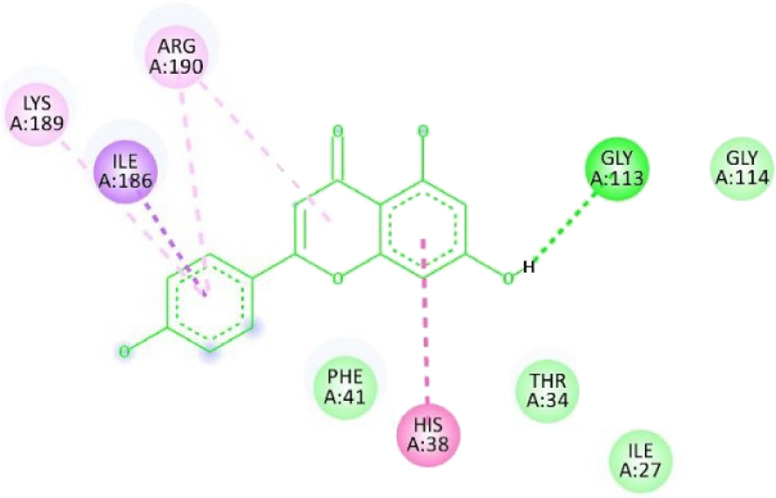
Leotulin	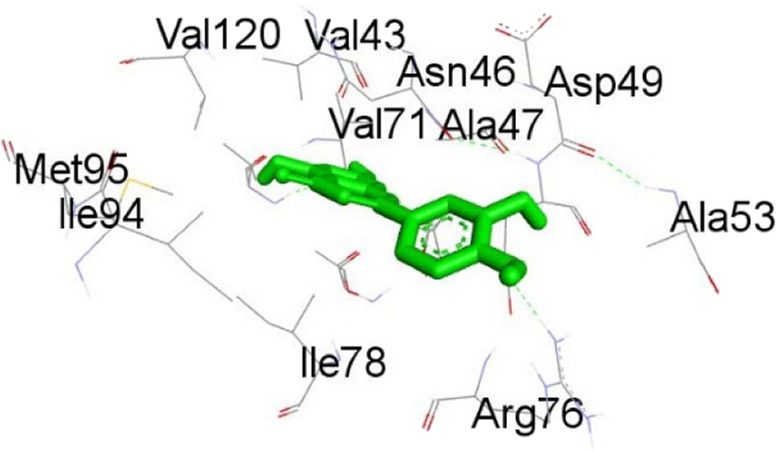	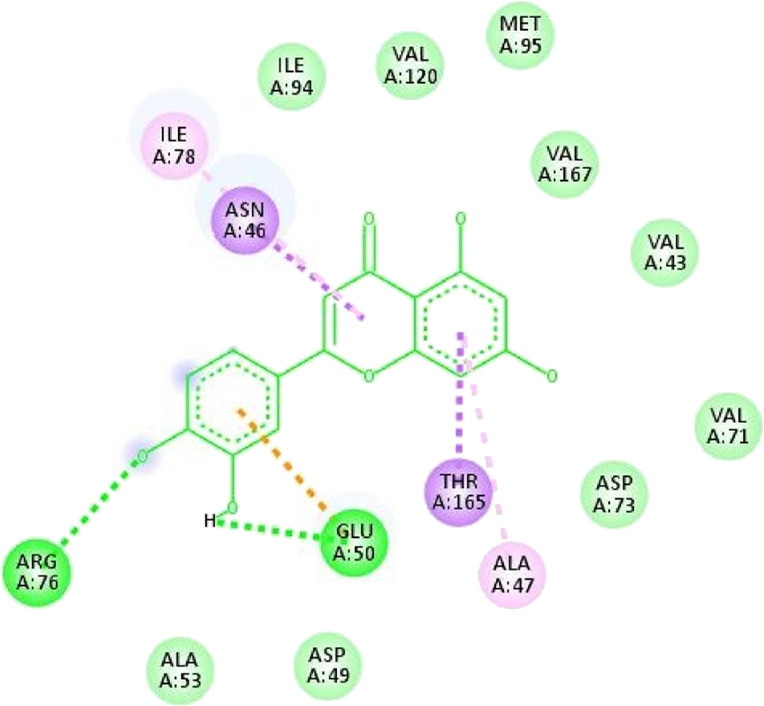

Supplementary Table S7 (Supplementary provided) lists the interaction of various ligands with the major components of *M. vulgare* extract and the antimicrobial protein target. Apigenin interacts with the antimicrobial protein target through hydrogen bonding and pi-bonding interactions with multiple amino acid residues, including GLY113, ILE186, HIS38, ARG190, LYS189, and ARG190. The distances between the ligand and the amino acid residues range from 1.8454 to 5.34399 Å. Biotin interacts with the antimicrobial protein target through hydrogen bonding and hydrophobic interactions with amino acid residues GLY77, THR165, ASP73, ALA100, and ILE78. The distances between the ligand and the amino acid residues range from 2.32752 to 5.36772 Å. Caffeic Acid interacts with the antimicrobial protein target through hydrogen bonding and pi-bonding interactions with amino acid residues THR165, ASP73, GLY77, GLU50, and ILE78. The distances between the ligand and the amino acid residues range from 2.04553 to 3.81553 Å. Catechin interacts with the antimicrobial protein target through hydrogen bonding and hydrophobic interactions with amino acid residues ARG76, GLU50, THR165, and ALA47. The distances between the ligand and the amino acid residues range from 2.61913 to 5.25295 Å.

Leotulin interacts with the antimicrobial protein target through hydrogen bonding, pi-bonding interactions, and hydrophobic interactions with amino acid residues ARG76, GLU50, THR165, ASN46, ILE78, and ALA47. The distances between the ligand and the amino acid residues range from 2.56108 to 5.05026 Å. Maleic Acid interacts with the antimicrobial protein target through hydrogen bonding and hydrophobic interactions with amino acid residues HIS99 and ALA100. The distances between the ligand and the amino acid residues range from 2.33271 to 2.67941 Å. Salicylic Acid interacts with the antimicrobial protein target through hydrogen bonding and hydrophobic interactions with amino acid residues ASP73, VAL120, and VAL167. The distances between the ligand and the amino acid residues range from 2.39911 to 5.39453 Å.Vanillic Acid interacts with the antimicrobial protein target through hydrogen bonding, pi-bonding interactions, and hydrophobic interactions with amino acid residues THR165, ASP73, VAL71, ASP73, THR165, ALA47, VAL43, VAL71, VAL167, and ILE78. The distances between the ligand and the amino acid residues range from 1.97698 to 5.36435 Å. All type of interactions is also shown in Supplementary Table S8 (Supplementary provided) as 3D and 2D.

## 4 Conclusion

In conclusion, the outcomes of the current study showed that the *M. vulgare* aqueous extract significantly exerts antioxidant, antimitotic, and antimicrobial activities. As well as a strong anti-hyperglycemic effect and pancreatic α-amylase inhibitory effect. These effects are due to its richness of a diversity of phenolic compounds. These findings support the folkloric use of *M. vulgare* decoction in the prevention and management of illnesses like diabetes, colds, and flu, and can also be used in the management of diabetes oxidative stress, and infectious diseases. Also, molecular docking studies revealed the compounds as viable potential drug candidates and could be explored in the discovery of antimitotic, antidiabetic, and antimicrobial protein targets. However, further experimental studies are needed to further validate the results of this study.

## Data Availability

The raw data supporting the conclusion of this article will be made available by the authors, without undue reservation.

## References

[B1] 1AJ6 (2023). NOVOBIOCIN-RESISTANT mutant (R136H) of the N-terminal 24 kda fragment of dna gyrase B complexed with NOVOBIOCIN at 2.3 angstroms resolution. Available online: https//www.rcsb.org/1AJ6 (accessed on April 31, 2023).

[B2] 1XO2 (2023). Crystal structure of a human cyclin-dependent kinase 6 complex with a flavonol inhibitor, fisetin. Available online: https//www.rcsb.org/1XO2 (accessed on April 31, 2023). 10.1021/jm049353p 15689157

[B3] 4W93 (2023). Human pancreatic alpha-amylase in complex with montbretin A. Available online: https//www.rcsb.org/4W93 (accessed on April 31, 2023).

[B4] Accelrys Software Inc (2005). Discovery Studio visualizer, 2. San Diego, CA, USA: Accelrys Software Inc.

[B5] AFNOR (1977). Aliments des animaux. Dosage des cendres brute. France: NF, V18–V101.

[B6] AFNOR (1985). NF V03-402. Épices et aromates - Détermination de la teneur en eau - Méthode par entraînement. » Afnor EDITIONS.

[B7] AFNOR (1972). NF V05-113. Fruits, légumes et produits dérivés - minéralisation des matières organiques - Méthode par incinération ». Afnor EDITIONS.

[B8] Akther N. Shawl A. S. Sultana S. Chandan B. K. Akhter M. (2013). Hepatoprotective activity of Marrubium vulgare against paracetamol induced toxicity. J. Pharm. Res. 7, 565–570. 10.1016/j.jopr.2013.06.023

[B9] Al-Tohamy R. Ali S. S. Saad-Allah K. Fareed M. Ali A. El-Badry A. (2018). Phytochemical analysis and assessment of antioxidant and antimicrobial activities of some medicinal plant species from Egyptian flora. J. Appl. Biomed. 16, 289–300. 10.1016/j.jab.2018.08.001

[B10] Alami Merrouni I. Kharchoufa L. Bencheikh N. Elachouri M. (2021). Ethnobotanical profile of medicinal plants used by people of North-eastern Morocco: cross-cultural and historical approach (part I). Ethnobot. Res. Appl. 21, 1–45. 10.32859/ERA.21.34.1-45

[B11] Ali-Rachedi F. Meraghni S. Touaibia N. (2018). Quantitative analysis of phenolic compounds of an Algerian endemic Scabiosa Atropurpurea sub. Maritima L. J. Bull. Société R. Des. Sci. Liège 87, 13–21.

[B12] Amessis-Ouchemoukh N. Abu-Reidah I. M. Quirantes-Piné R. Madani K. Segura-Carretero A. (2014). Phytochemical profiling, *in vitro* evaluation of total phenolic contents and antioxidant properties of marrubium vulgare (horehound) leaves of plants growing in Algeria. Ind. Crops Prod. 61, 120–129. 10.1016/j.indcrop.2014.06.049

[B13] Bissel S. J. Winkler C. C. Deltondo J. Wang G. Williams K. Wiley C. A. (2014). Coxsackievirus B4 myocarditis and meningoencephalitis in newborn twins. Neuropathology 34, 429–437. 10.1111/neup.12121 24702280 PMC4188796

[B14] Barros L. Cabrita L. Boas M. V. Carvalho A. M. Ferreira I. C. F. R. (2011). Chemical, biochemical and electrochemical assays to evaluate phytochemicals and antioxidant activity of wild plants. Food Chem. 127, 1600–1608. 10.1016/j.foodchem.2011.02.024

[B15] Bencheikh N. Bouhrim M. Merrouni I. A. Boutahiri S. Legssyer A. Elachouri M. (2021). Antihyperlipidemic and antioxidant activities of flavonoid-rich extract of ziziphus lotus (L) lam fruits. Appl. Sci. 11, 7788. 10.3390/app11177788

[B16] Bencheikh N. Elachouri M. Subhash C., M. (2022a). Ethnobotanical, pharmacological, phytochemical, and clinical investigations on Moroccan medicinal plants traditionally used for the management of renal dysfunctions. J. Ethnopharmacol. 292, 115178. 10.1016/j.jep.2022.115178 35278608

[B17] Bencheikh N. Ouahhoud S. Cordero M. A. W. Alotaibi A. Fakchich J. Ouassou H. (2022b). Nephroprotective and Antioxidant Effects of Flavonoid-Rich Extract of Thymelaea microphylla Coss. et Dur Aerial Part. Appl. Sci. 12, 9272. 10.3390/app12189272

[B18] Benzidane N. Smahi R. Zabouche B. Abdelhalim Makrouf L. A. Arrar L. (2020). Phytochemical study and antimicrobial activity of Algerian Marrubium vulgare leaf and stem extracts. J. Drug Deliv. Ther. 10, 70–74. 10.22270/jddt.v10i5.4353

[B19] Bergeron L. (1995). Effet de la Teneur en Eau du Sol Sur le Rendement et la Qualité Des Fruits du Bleuet Nain; Université du Québec à Chicoutimi: Chicoutimi. QC, Canada. 978-1-4123-0614-0.

[B20] Boudjelal A. Henchiri C. Sari M. Sarri D. Hendel N. Benkhaled A. (2013). Herbalists and wild medicinal plants in M’Sila (North Algeria): an ethnopharmacology survey. J. Ethnopharmacol. 148, 395–402. 10.1016/j.jep.2013.03.082 23643544

[B21] Bouhrim M. Ouassou H. Boutahiri S. Daoudi N. E. Mechchate H. Gressier B. (2021). Opuntia dillenii (ker gawl) haw., seeds oil antidiabetic potential using *in vivo*, *in vitro*, *in situ*, and *ex vivo* approaches to reveal its underlying mechanism of action. Molecules 26, 1677. 10.3390/molecules26061677 33802826 PMC8002680

[B22] Bouterfas K. Mehdadi Z. Elaoufi M. M. Aouad L. Latreche A. Benchiha W. (2018). *In vitro* antibacterial activity of flavonoids extracts from three Algerian horehound (Marrubium vulgare L) leaves) leaves Orient. Pharm. Exp. Med. 18, 59–66. 10.1007/s13596-017-0287-5

[B23] Bouterfas K. Mehdadi Z. Elaoufi M. M. Latreche A. Benchiha W. (2016). Antioxidant activity and total phenolic and flavonoids content variations of leaves extracts of white Horehound (Marrubium vulgare Linné) from three geographical origins. Ann. Pharm. Fr. 74, 453–462. 10.1016/j.pharma.2016.07.002 27553439

[B24] Bouyahya A. El Omari N. Elmenyiy N. Guaouguaou F. E. Balahbib A. Belmehdi O. (2021). Moroccan antidiabetic medicinal plants: Ethnobotanical studies, phytochemical bioactive compounds, preclinical investigations, toxicological validations and clinical evidences; challenges, guidance and perspectives for future management of diabetes worldwide. Trends Food Sci. Technol. 115, 147–254. 10.1016/j.tifs.2021.03.032

[B25] Braca A. Politi M. Sanogo R. Sanou H. Morelli I. Pizza C. (2003). Chemical composition and antioxidant activity of phenolic compounds from wild and cultivated sclerocarya birrea (anacardiaceae) leaves. J. Agric. Food Chem. 51, 6689–6695. 10.1021/jf030374m 14582961

[B26] CambridgeSoft (2009b). Chem 3D Pro 12.0 (copyright) 1986 to 2009. Cambridge, MA, USA: CambridgeSoft Corp.

[B27] CambridgeSoft (2009a). Ultra 12.0 0 (copyright) 1986 to 2009. Cambridge, MA, USA: CambridgeSoft Corp.

[B28] Chavan U. Shahidi F. Naczk M. (2001). Extraction of condensed tannins from beach pea (Lathyrus maritimus L) as affected by different solvents) as affected by different solvents. Food Chem. 75, 509–512. 10.1016/s0308-8146(01)00234-5

[B29] Costamagna M. S. Zampini I. C. Alberto M. R. Cuello S. Torres S. Pérez J. (2016). Polyphenols rich fraction from Geoffroea decorticans fruits flour affects key enzymes involved in metabolic syndrome, oxidative stress and inflammatory process. Food Chem. 190, 392–402. 10.1016/j.foodchem.2015.05.068 26212988

[B30] Crozier A. Jaganath I. B. Clifford M. N. (2009). Dietary phenolics: Chemistry, bioavailability and effects on health. Nat. Prod. Rep. 26, 1001–1043. 10.1039/b802662a 19636448

[B31] Daoudi N. E. Bouhrim M. Ouassou H. Legssyer A. Mekhfi H. Ziyyat A. (2020). Inhibitory effect of roasted/unroasted Argania spinosa seeds oil on α-glucosidase, α-amylase and intestinal glucose absorption activities. South Afr. J. Bot. 135, 413–420. 10.1016/j.sajb.2020.09.020

[B32] De Oliveira Pedro R. Takaki M. Gorayeb T. C. C. Bianchi V. L. D. Thomeo J. C. Tiera M. J. (2013). Synthesis, characterization and antifungal activity of quaternary derivatives of chitosan on Aspergillus flavus. Microbiol. Res. 168, 50–55. 10.1016/j.micres.2012.06.006 22819383

[B33] Deepthi B. Sowjanya K. Lidiya B. Bhargavi R. Babu P. (2017). A modern review of diabetes mellitus: an annihilatory metabolic disorder. J. Silico Vitr. Pharmacol. 3, 1–5. 10.21767/2469-6692.100014

[B34] Dib K. Cherrah Y. Rida S. Filali-Maltouf A. Ennibi O. (2021). *In vitro* antibacterial activity of myrtus communis L. And marrubium vulgare L. Leaves against aggregatibacter actinomycetemcomitans and eikenella corrodens. Altern. Med. 2021, 1–8. 10.1155/2021/8351332 PMC8548106 34712349

[B35] Djahra A. B. Bordjiba O. Benkherara S. (2013). Extraction, séparationet activité antibactérienne des tanins de marrube blanc (Marrubium vulgare L). Phytotherapie 11, 348–352. 10.1007/s10298-013-0819-1

[B36] Dra L. A. Sellami S. Rais H. Aziz F. Aghraz A. Bekkouche K. (2019). Antidiabetic potential of Caralluma europaea against alloxan-induced diabetes in mice. Saudi J. Biol. Sci. 26, 1171–1178. 10.1016/j.sjbs.2018.05.028 31516346 PMC6733698

[B37] Duarte M. C. T. Leme E. E. Delarmelina C. Soares A. A. Figueira G. M. Sartoratto A. (2007). Activity of essential oils from Brazilian medicinal plants on *Escherichia coli* . J. Ethnopharmacol. 111, 197–201. 10.1016/j.jep.2006.11.034 17210236

[B38] Dwek R. A. Butters T. D. Platt F. M. Zitzmann N. (2002). Targeting glycosylation as a therapeutic approach. Nat. Rev. Drug Discov. 1, 65–75. 10.1038/nrd708 12119611

[B39] Eberhardt J. Santos-Martins D. Tillack A. F. Forli S. (2021). AutoDock Vina 1.2.0: New docking methods, expanded force field, and Python bindings. J. Chem. Inf. Model. 61, 3891–3898. 10.1021/acs.jcim.1c00203 34278794 PMC10683950

[B40] Ekor M. (2014). The growing use of herbal medicines: issues relating to adverse reactions and challenges in monitoring safety. Front. Pharmacol. 4, 177–210. 10.3389/fphar.2013.00177 24454289 PMC3887317

[B41] Elberry A. A. Harraz F. M. Ghareib S. A. Gabr S. A. Nagy A. A. Abdel-Sattar E. (2015). Methanolic extract of Marrubium vulgare ameliorates hyperglycemia and dyslipidemia in streptozotocin-induced diabetic rats. Int. J. Diabetes Mellit. 3, 37–44. 10.1016/j.ijdm.2011.01.004

[B42] Fakchich J. Elachouri M. (2021). An overview on ethnobotanico-pharmacological studies carried out in Morocco, from 1991 to 2015: Systematic review (part 1). J. Ethnopharmacol. 267, 113200–200. 10.1016/j.jep.2020.113200 32750461

[B43] Fayyad A. G. S. Ibrahim N. Yaakob W. A. (2014). Phytochemical screening and antiviral activity of Marrubium vulgare. J. Microbiol. 10, 106–111.

[B44] Ferreira L. G. Dos Santos R. N. Oliva G. Andricopulo A. D. (2015). Molecular docking and structure-based drug design strategies. Molecules 20, 13384–13421. 10.3390/molecules200713384 26205061 PMC6332083

[B45] Ghedadba N. Bousselsela H. Hambaba L. Benbia S. Mouloud Y. (2014). Évaluation de l’activité antioxydante et antimicrobienne des feuilles et des sommités fleuries de Marrubium vulgare L. Phytotherapie 12, 15–24. 10.1007/s10298-014-0832-z

[B46] Hanhineva K. Törrönen R. Bondia-Pons I. Pekkinen J. Kolehmainen M. Mykkänen H. (2010). Impact of dietary polyphenols on carbohydrate metabolism. Int. J. Mol. Sci. 11, 1365–1402. 10.3390/ijms11041365 20480025 PMC2871121

[B47] He Q. Lv Y. Yao K. (2007). Effects of tea polyphenols on the activities of α-amylase, pepsin, trypsin and lipase. Food Chem. 101, 1178–1182. 10.1016/j.foodchem.2006.03.020

[B95] Hosseinzadeh H. Nassiri-Asl M. (2015). Pharmacological effects of Glycyrrhiza spp. and its bioactive constituents: update and review. Phytother. Res. 29 (12), 1868–1886. 10.1002/ptr.5487 26462981

[B48] Kakoma P. K. Kadiebwe D. M. Kayembe A. M. Kashindi P. M. Bugeme M. Mukuku O. (2014). Diabetic ketoacidosis in adults in sendwe hospital lubumbashi: about 51 cases. Pan Afr. Med. J. 17, 324–325. 10.11604/pamj.2014.17.324.3545 25328619 PMC4198266

[B49] Karryev M. Bairyev C. Ataeva A. (1976). Some therapeutic properties and phytochemistry of common horehound. J. Seriya Biol. Nauk. 3, 86–88.

[B50] Khanbabaee K. van Ree T. (2001). Tannins: classification and definition. Nat. Prod. Rep. 18, 641–649. 10.1039/b101061l 11820762

[B51] Khatware K. Annapurna A. (2014). The effect of Quercetin on blood glucose levels of normal and streptozotocin induced diabetic (type I & type ii) rats. J. Pharm. Chem. Biol. Sci. 4.

[B52] Koncic M. Z. Kremer D. Karlovic K. Kosalec I. (2010). Evaluation of antioxidant activities and phenolic content of Berberis vulgaris L. and Berberis croatica Horvat. Food Chem. Toxicol. J. 48, 2176–2180. 10.1016/j.fct.2010.05.025 20488218

[B53] Kotan R. Kordali S. Cakir A. Kesdek M. Kaya Y. Kilic H. (2008). Antimicrobial and insecticidal activities of essential oil isolated from Turkish Salvia hydrangea DC. ex Benth. Ex. Benth. Biochem. Syst. Ecol. 36, 360–368. 10.1016/j.bse.2007.12.003

[B54] Kroeze D. (2020). Le pH l’acidité: L’effet de l’acidité sur vos plantes. Canna Res.

[B55] Kumar V. Singhalb A. (n.d.). Germinating seeds of the Mung bean, Vigna radiata (Fabaceae), as a model for the preliminary evaluation of cytotoxic effects of drugs. J. Biocell. 33, 19–24. 19499882

[B56] Kumari S. Kumar P. Wanjari M. Palani S. (2012). Antidiabetic activity of Pandanus fascicularis Lamk - aerial roots in alloxan-induced hyperglycemic rats. Int. J. Nutr. Pharmacol. Neurol. Dis. 2, 105. 10.4103/2231-0738.95943

[B57] Kurbatova N. V. Muzychkina R. A. Mukhitdinov N. M. Parshina G. N. (2003). Comparative phytochemical investigation of the composition and content of biologically active substances in Marrubium vulgare and M. alternidens. Chem. Nat. Compd. 39, 501–502. 10.1023/B:CONC.0000011128.64886.f4

[B58] Ladoh Yemeda C. F. Dibong S. D. Nyegue M. A. Djembissi Talla R. Mpondo E. Yinyang J. (2014). Activité antioxydante des extraits méthanoliques de Phragmanthera capitata (Loranthaceae) récoltée sur Citrus sinensis. J. Appl. Biosci. 84, 7636–7643.

[B59] Loizzo M. R. Leporini M. Sicari V. Falco T. Pellicanò T. M. Tundis R. (2017). Investigating the *in vitro* hypoglycaemic and antioxidant properties of Citrus × clementina Hort. juice. Juice. Eur. Food Res. Technol. 12, 523–534. 10.1007/s00217-017-2978-z

[B60] Lorrain B. Chira K. Teissedre P. L. (2011). Phenolic composition of merlot and cabernet-sauvignon grapes from bordeaux vineyard for the 2009-vintage: comparison to 2006, 2007 and 2008 vintages. Food Chem. 126, 1991–1999. 10.1016/j.foodchem.2010.12.062 25213988

[B61] Martel J. Ojcius D. M. Chang C. J. Lin C. S. Lu C. C. Ko Y. F. (2017). Anti-obesogenic and antidiabetic effects of plants and mushrooms. J. Nat. Rev. Endocrinol. 13, 149–160. 10.1038/nrendo.2016.142 27636731

[B62] Masoodi M. Ali Z. Liang S. Yin H. Wang W. Khan I. A. (2015). Labdane diterpenoids from Marrubium vulgare. Phytochem. Lett. 13, 275–279. 10.1016/j.phytol.2015.07.005

[B63] Matkowski A. Tasarz P. Szypuła E. (2008). Antioxidant activity of herb extracts from five medicinal plants from Lamiaceae, subfamily Lamioideae. J. Med. Plants Res. 2, 321–330.

[B64] Mbayo M. K. Kalonda E. M. Muya R. K. Tshisand P. T. Kanangila A. B. Maseho F. M. (2016). Test d’activité antimitotique et étude chimique préliminaire de quelques Euphorbiaceae du Katanga méridional (RDC). Phytotherapie 2012, 1–13. 10.1007/s10298-016-1060-5

[B65] Meyre-Silva C. Cechinel-Filho V. (2010). A review of the chemical and pharmacological aspects of the genus marrubium. Curr. Pharm. Des. 16, 3503–3518. 10.2174/138161210793563392 20942795

[B66] Molina-Salinas G. M. Ramos-Guerra M. C. Vargas-Villarreal J. Mata-Cárdenas B. D. Becerril-Montes P. Said-Fernández S. (2006). Bactericidal activity of organic extracts from flourensia cernua DC against strains of *Mycobacterium tuberculosis* . Mycobacterium Tuberc. Arch. Med. Res. 37, 45–49. 10.1016/j.arcmed.2005.04.010 16314185

[B67] Morris G. M. Huey R. Lindstrom W. Sanner M. F. Belew R. K. Goodsell D. S. (2009). AutoDock4 and AutoDockTools4: automated docking with selective receptor flexibility. J. Comput. Chem. 30, 2785–2791. 10.1002/jcc.21256 19399780 PMC2760638

[B68] Musa Özcan M. (2006). Determination of the mineral compositions of some selected oil-bearing seeds and kernels using Inductively Coupled Plasma Atomic Emission Spectrometry (ICP-AES). Grasas Aceites 57, 211–218. 10.3989/gya.2006.v57.i2.39

[B69] Olmedilla B. Granado F. Gil-Martinez E. Blanco I. Rojas-Hidalgo E. (1997). Reference values for retinol, tocopherol, and main carotenoids in serum of control and insulin-dependent diabetic Spanish subjects. Clin. Chem. 43, 1066–1071. 10.1093/clinchem/43.6.1066 9191562

[B70] Onesti C. E. Vicier C. André F. (2015). Qu'attendre de la génomique à haut débit dans les cancers du sein métastatiques ? Breast 24, S19–S22. 10.1016/j.breast.2015.07.006 26238439

[B72] Ouassou H. Bouhrim M. Bencheikh N. Addi M. Hano C. Mekhfi H. (2021). *In vitro* antioxidant properties, glucose-diffusion effects, α-amylase inhibitory activity, and antidiabetogenic effects of C. Europaea extracts in experimental animals. Antioxidants 10, 1747. 10.3390/antiox10111747 34829618 PMC8614910

[B73] Ouedraogo S. Yoda J. Traore T. K. Nitiema M. Sombie B. C. Diawara H. Z. (2021). Production de matières premières et fabrication des médicaments à base de plantes médicinales. Int. J. Biol. Chem. Sci. 15, 750–772. 10.4314/ijbcs.v15i2.28

[B75] Porto C. D. Porretto E. Decorti D. (2013). Ultrasonics Sonochemistry Comparison of ultrasound-assisted extraction with conventional extraction methods of oil and polyphenols from grape (Vitis vinifera L) seeds. Ultrason. - Sonochemistry 20, 1076–1080. 10.1016/j.ultsonch.2012.12.002 23305938

[B76] Prieto P. Pineda M. Aguilar M. (1999). Spectrophotometric quantitation of antioxidant capacity through the formation of a phosphomolybdenum complex: specific application to the determination of vitamin E. Anal. Biochem. 269, 337–341. 10.1006/abio.1999.4019 10222007

[B77] Pukalskas A. Venskutonis P. R. Salido S. Waard P. van Beek T. A. (2012). Isolation, identification and activity of natural antioxidants from horehound (Marrubium vulgare L) cultivated in Lithuania) cultivated in Lithuania. Food Chem. 130, 695–701. 10.1016/j.foodchem.2011.07.112

[B78] Rezgui M. Majdoub N. Mabrouk B. Baldisserotto A. Bino A. Ben Kaab L. B. (2020). Antioxidant and antifungal activities of marrubiin, extracts and essential oil from Marrubium vulgare L. against pathogenic dermatophyte strains. J. Mycol. Med. 30, 100927. 10.1016/j.mycmed.2020.100927 31983544

[B79] Sartoratto A. Machado A. L. M. Delarmelina C. Figueira G. M. Duarte M. C. T. Rehder V. L. G. (2004). Composition and antimicrobial activity of essential oils from aromatic plants used in Brazil. Braz. J. Microbiol. 35, 275–280. 10.1590/S1517-83822004000300001

[B80] Singh J. Singh J. Sharma D. (2018). Traditional wisdom to treat the most common ailments in chopal region of Shimla district, Himachal Pradesh, India. Plant Arch. 18, 2759–2769.

[B81] Sisodia D. Sisodia D. S. (2018). Prediction of diabetes using classification algorithms. Procedia Comput. Sci. 132, 1578–1585. 10.1016/j.procs.2018.05.122

[B83] Taib M. Rezzak Y. Bouyazza L. Lyoussi B. (2020). Medicinal uses, phytochemistry, and pharmacological activities of quercus species. Evidence-based complement. Altern. Med. 2020, 20. 10.1155/2020/1920683 PMC7415107 32802116

[B84] Tamert A. Latreche A. Aouad L. (2017). Criblage phytochimique et activité antimicrobienne des extraits de Thymus serpyllum et de Thymus vulgaris du mont de Tessala (Algérie occidentale). Phytotherapie 15, 384–394. 10.1007/s10298-017-1132-1

[B85] Tchoumtchoua J. Mouchili O. R. Ateba S. B. Zingue S. Halabalaki M. Mbanya J. C. (2014). Safety assessment of the methanol extract of the stem bark of Amphimas pterocarpoides Harms: acute and subchronic oral toxicity studies in Wistar rats. Toxicol. Rep. 1, 877–884. 10.1016/j.toxrep.2014.10.003 28962299 PMC5598524

[B86] Trott O. Olson A. J. (2010). AutoDock Vina: improving the speed and accuracy of docking with a new scoring function, efficient optimization, and multithreading. J Comput. Chem. 31 (2), 455–461. 10.1002/jcc.21334 19499576 PMC3041641

[B87] Wang H. Wang J. Qiu C. Ye Y. Guo X. Chen G. (2017). Comparison of phytochemical profiles and health benefits in fiber and oil flaxseeds (Linum usitatissimum L). Food Chem. 214, 227–233. 10.1016/j.foodchem.2016.07.075 27507470

[B88] Wojdyło A. Oszmiański J. Czemerys R. (2007). Antioxidant activity and phenolic compounds in 32 selected herbs. Food Chem. 105, 940–949. 10.1016/j.foodchem.2007.04.038

[B89] Xiaomei M. Herbert Y. (2006). Global burden of cancer. Yale J. Biol. Med. 79, 85–94. 17940618 PMC1994799

[B90] Yousefi K. Fathiazad F. Soraya H. Rameshrad M. Maleki-Dizaji N. Garjani A. (2014). Marrubium vulgare L. methanolic extract inhibits inflammatory response and prevents cardiomyocyte fibrosis in isoproterenol-induced acute myocardial infarction in rats. BioImpacts 4, 21–27. 10.5681/bi.2014.001 24790895 PMC4005279

[B91] Yousefi K. Soraya H. Fathiazad F. Khorrami A. Hamedeyazdan S. Maleki-Dizaji N. (2013). Cardioprotective effect of methanolic extract of Marrubium vulgare L. on isoproterenol-induced acute myocardial infarction in rats. Indian J. Exp. Biol. 51, 653–660. https://nopr.niscpr.res.in/handle/123456789/20478 24228389

[B92] Yuan H. Ma Q. Ye L. Piao G. (2016). The traditional medicine and modern medicine from natural products. Molecules 21, 559. 10.3390/molecules21050559 27136524 PMC6273146

[B93] Yusuf D. Davis A. M. Kleywegt G. J. Schmitt S. (2008). An alternative method for the evaluation of docking performance: RSR vs. RMSD. J. Chem. Inf. Model. 48, 1411–1422. 10.1021/ci800084x 18598022

[B94] Zentgraf M. Steuber H. Koch C. La Motta C. Sartini S. Sotriffer C. A. (2007). How reliable are current docking approaches for structure-based drug design? Lessons from aldose reductase. Angew. Chem. Int. Ed. 46, 3575–3578. 10.1002/anie.200603625 17394265

