# Supplementary material for: Insight into biological activities of chemically characterized extract from Marrubium vulgare L. in vitro, in vivo and in silico approaches
Source: Front Chem. 2023 Aug 17;11:1238346. doi: 10.3389/fchem.2023.1238346 (PMC10470090; doi:10.3389/fchem.2023.1238346)
Supplement: Supplementary file 1 [file DataSheet1.docx]

**Insight into biological activities of chemically characterized extract from *Marrubium vulgare* L. *In vitro, in vivo*  and *in silico* approaches**

**Aman Allah Gourich^1^, Hanane Touijer^1^, Aziz Drioiche^1^, Ayoub Asbabou^1^, Firdaous Remok^1^, Soukaina Saidi^1^, Atika Ailli^1^, Farhan Siddique^2^, Mohammed Bourhia^3^,** **Ahmad Mohammad Salamatulla^4^, Lahcen Ouahmane^5^, Aicha Mouradi^1^, Bruno Eto^6^, Touriya Zair^1^***

^1^Research Team of Chemistry of Bioactive Molecules and the Environment, Laboratory of Innovative Materials and Biotechnology of Natural Resources, Faculty of Sciences, Moulay Ismaïl University, B.P.11201 Zitoune, Meknes 50070, Morocco.

^2^Laboratory of Organic Electronics, Department of Science and Technology, Linköping University,

SE-60174 Norrköping, Sweden

^3^Department of Chemistry and Biochemistry, Faculty of Medicine and Pharmacy, Ibn Zohr University, Laayoune 70000, Morocco

^4^Department of Food Science & Nutrition, College of Food and Agricultural Sciences, King Saud University, 11 P.O. Box 2460, Riyadh 11451, Saudi Arabia

^5^Laboratory of Microbial Biotechnologies, Agrosciences and Environment (BioMAgE), Labeled Research Unit-CNRSTN°4, Cadi Ayyad University, Marrakesh 40000, Morocco

^6^Laboratoires TBC, Laboratory of Pharmacology, Pharmacokinetics and Clinical Pharmacy, Faculty of Pharmacy, University of Lille, 3, Rue du Professeur Laguesse, B.P. 83, F-59000 Lille, France.

*Correspondence: author: [.zair@umi.ac.ma](mailto:.zair@umi.ac.ma) (TZ); mohabourhi@gmail.com (MB)

Supplementary Material

**
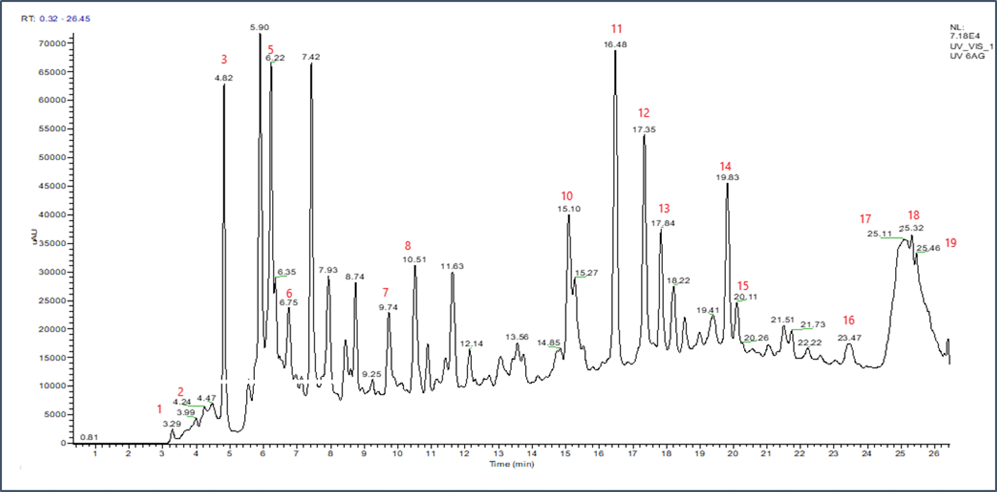
**

Figure 1S. Chromatogram of the aqueous extract of *M. vulgare* leaves, revealing the presence of 19 chemical compounds identified by HPLC/UV-ESI-MS analysis.

Table 2S. Molecular docking binding energies in kcal/mol of major components of *M. vulgare* extract ligands with antimitotic (1XO2), antidiabetic (4W93) and antimicrobial (1AJ6) protein targets.

| Ligand Name | 1XO2  Binding Energy (kcal/mol) | 4W93  Binding Energy (kcal/mol) | 1AJ6  Binding Energy (kcal/mol) |
| --- | --- | --- | --- |
| Apigenin | -6.8 | -8.3 | -7.0 |
| Biotin | -5.8 | -5.7 | -5.2 |
| Caffeic Acid | -6.9 | -6.5 | -6.0 |
| Catechin | -6.6 | -8.2 | -6.8 |
| Leotulin | -9.3 | -8.7 | -7.2 |
| Maleic Acid | -4.5 | -4.5 | -4.3 |
| Salicylic acid | -5.9 | -5.6 | -5.8 |
| Vanillic Acid | -6.2 | -5.5 | -5.5 |

Table 3S. Interaction of antimitotic protein target with the major components of *M. vulgare* extract ligands representing the binding pocket residue amino acids with distances and type of interacting bonds.

| Ligands with 1XO2  antimitotic protein | Interacting  Amino Acid Residues | Interacting Amino Acid Residues Distance in Angstrom | Types of Bonding Interactions |
| --- | --- | --- | --- |
| Apigenin | GLU52  ARG46  GLN48  LEU56  TYR24  PRO55  PRO55 | 2.79898  1.97679  2.77496  3.99331  4.97416  3.96852  4.29262 | H-bond  H-bond  H-bond  π – bond  alkyl-bond  π – bond  alkyl-bond |
| Biotin | VAL27  VAL27  LYS43  ALA162  ALA162  PHE98 | 4.83687  4.78311  4.89912  4.19142  5.4398  4.7349 | alkyl-bond  alkyl-bond  alkyl-bond  alkyl-bond  hydrophobic  alkyl-bond |
| Caffeic Acid | GLU61  VAL101  PHE98  VAL27  LYS43  ALA162 | 2.56413  2.69882  4.74682  4.96971  5.24013  4.01289 | H-bond  H-bond  alkyl-bond  alkyl-bond  hydrophobic  alkyl-bond |
| Catechin | GLN48  ARG46  GLY53  PRO55  ARG46 | 2.62557  2.15801  2.53691  4.34054  5.32928 | H-bond  H-bond  H-bond  alkyl-bond  hydrophobic |
| Leotulin | GLU21  ASP163  H - O  ASP104  ILE19  ILE19  VAL27  LEU152  VAL27  LEU152 | 2.91878  2.05529  2.15239  2.61701  3.57673  3.31492  3.4841  3.65281  4.87555  4.95756 | H-bond  H-bond  H-bond  H-bond  π – bond  π – bond  π – bond  π – bond  alkyl-bond  alkyl-bond |
| Maleic Acid | ASP163  GLU99  H - O | 2.10569  2.86735  2.67391 | H-bond  H-bond  H-bond |
| Salicylic acid | LYS43  ASP163  H - O  GLU61  PHE98  VAL27  ALA41  VAL77  LEU152  ALA162 | 2.49075  2.53593  2.3432  2.51861  4.92363  5.08407  4.62416  5.44102  5.11759  4.11462 | H-bond  H-bond  H-bond  H-bond  π – bond  hydrophobic  π – bond  hydrophobic  hydrophobic  π – bond |
| Vanillic Acid | ASP163  GLU61  ASP163  ALA162  VAL27  VAL27  ALA41  VAL77  LEU152 | 2.24252  2.91779  3.38009  3.83442  4.3061  5.23266  5.11258  5.34323  5.31901 | H-bond  H-bond  π – bond  π – bond  alkyl-bond  hydrophobic  hydrophobic  hydrophobic  hydrophobic |

Table 4S .Interaction of antimitotic protein target with the major components of *M. vulgare* extract ligands showing the 3D and 2D structural view.

| **Ligands with 1XO2** | **3D Amino Acid Interactions View** | **2D Amino Acid Interactions View** |
| --- | --- | --- |
| **Apigenin** | 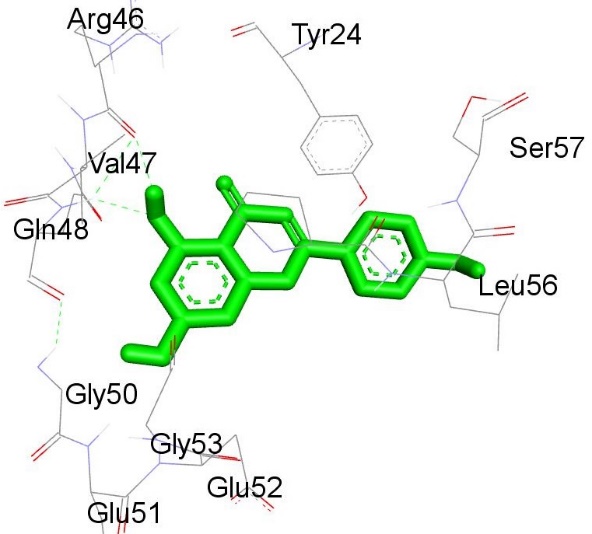 | 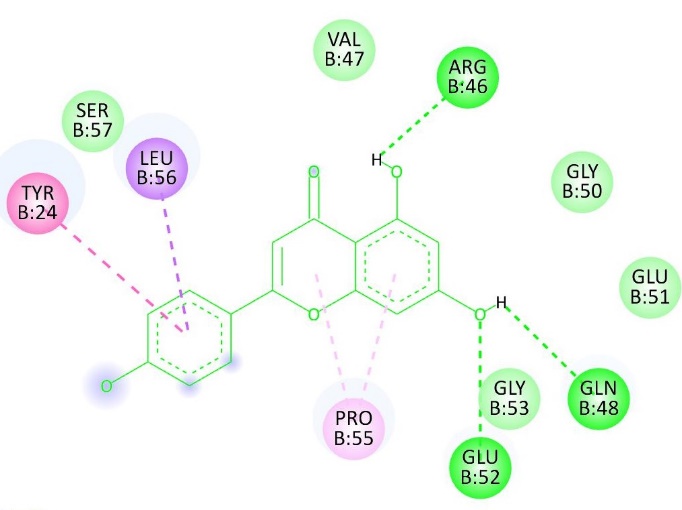 |
| **Biotin** | 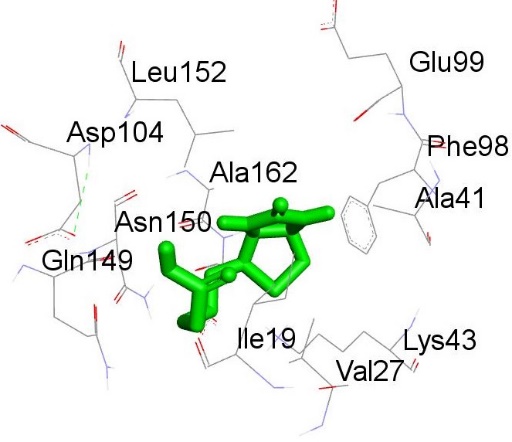 | 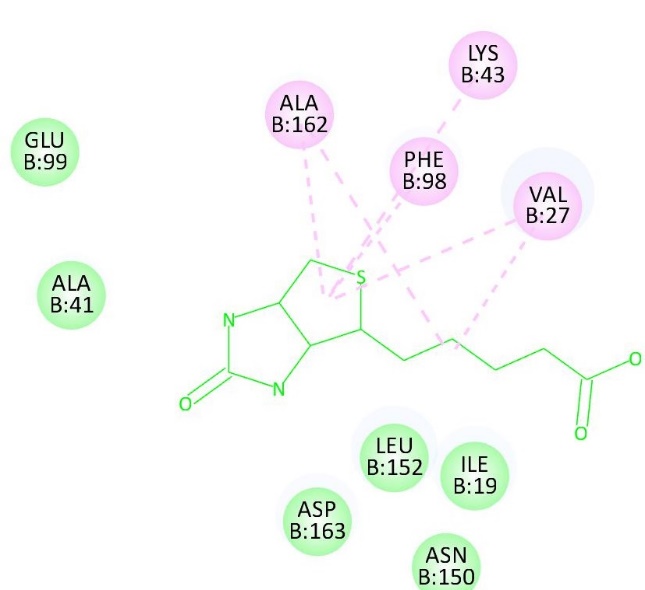 |
| **Caffeic acid** | 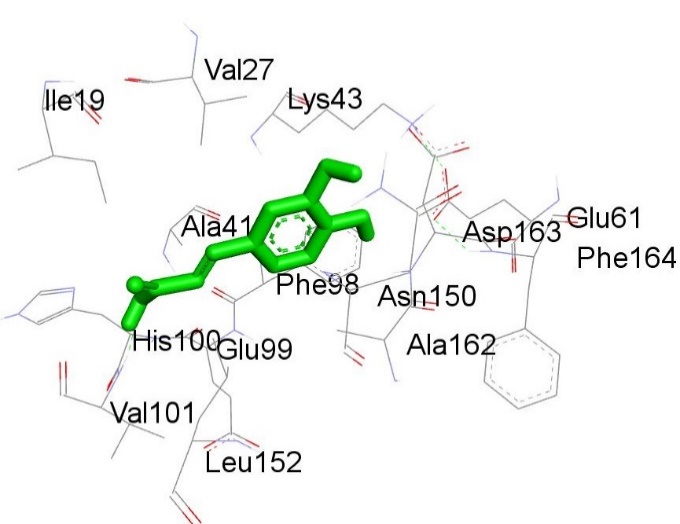 | 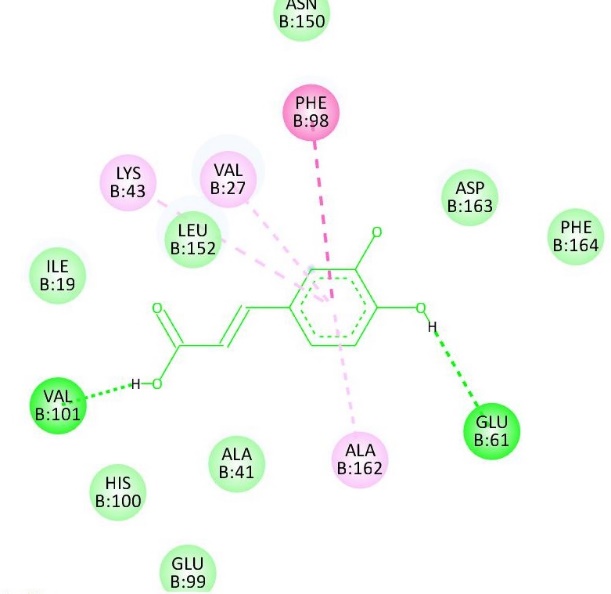 |
| **Catechin** | 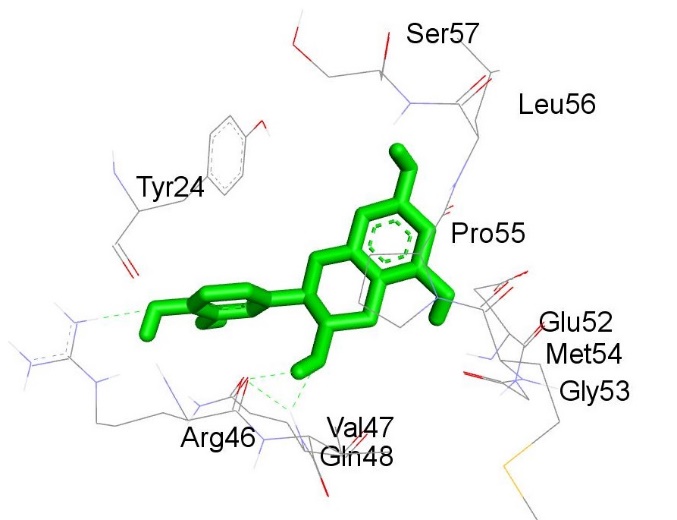 | 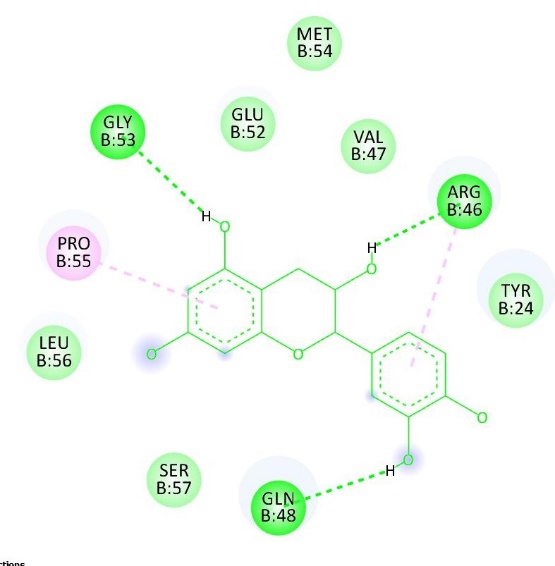 |
| **Leotulin** | 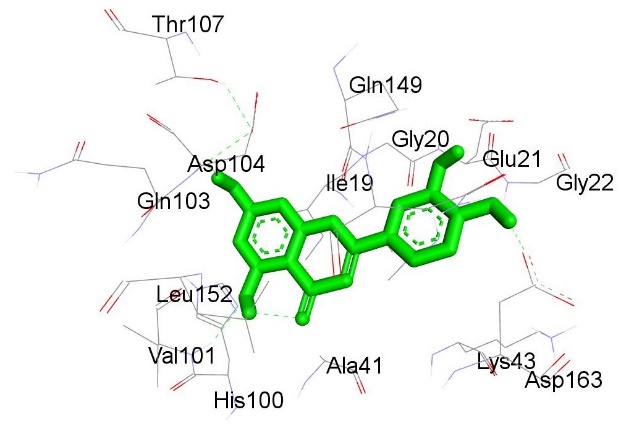 | 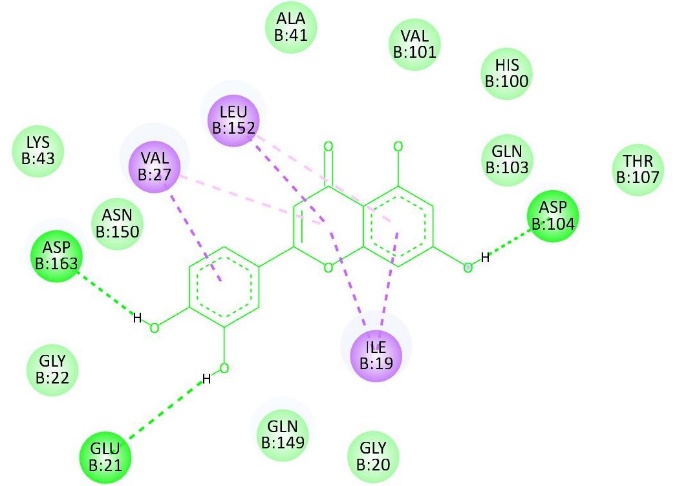 |
| **Maleic acid** | 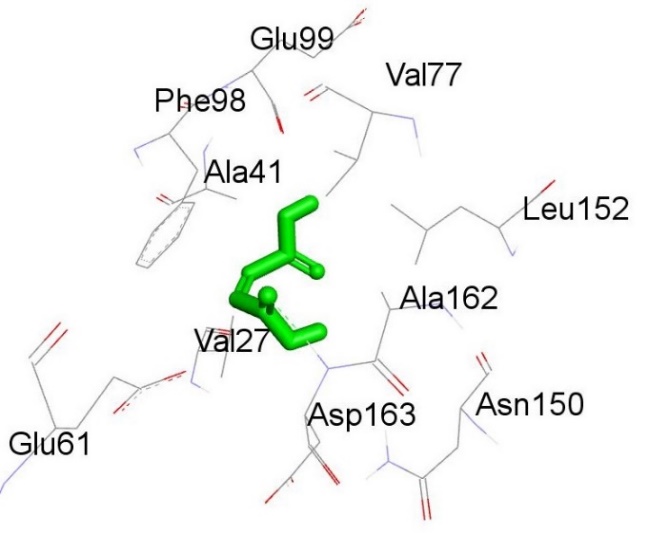 | 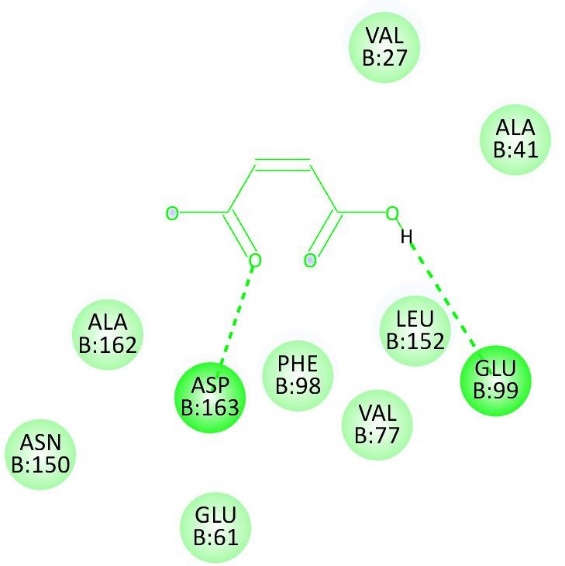 |
| **Salicylic acid** | 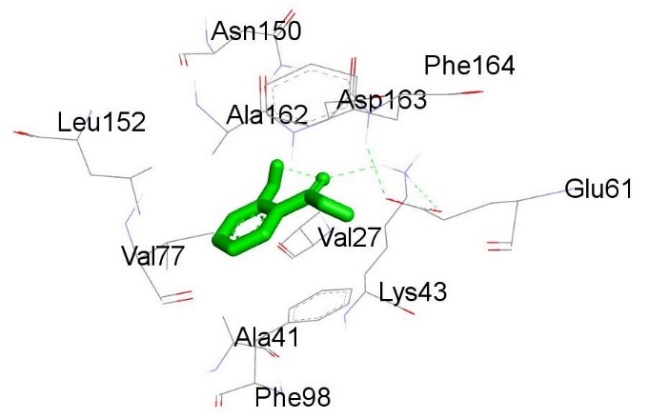 | 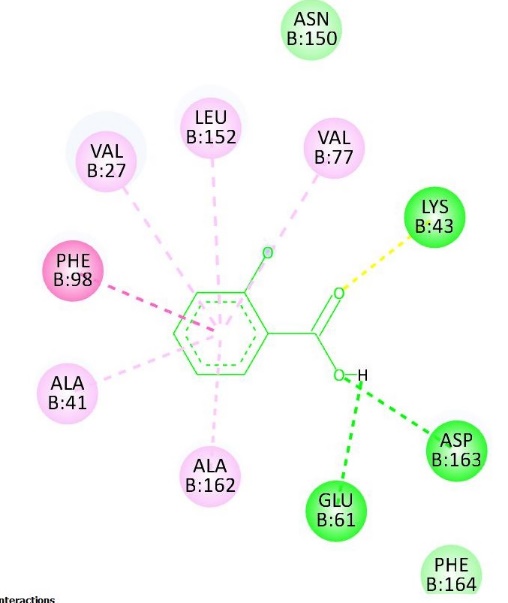 |
| **Vanillic acid** | 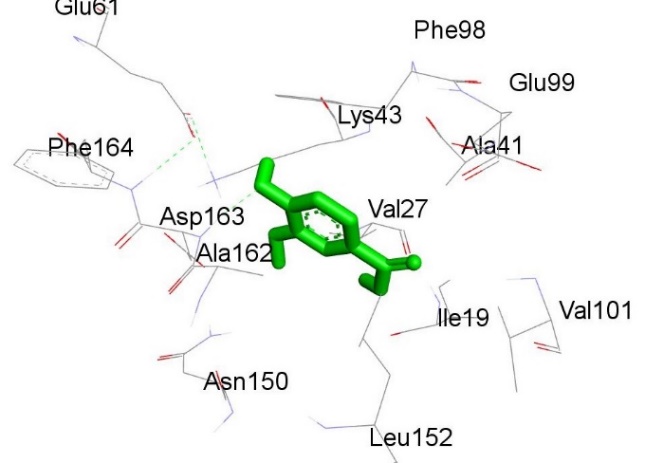 | 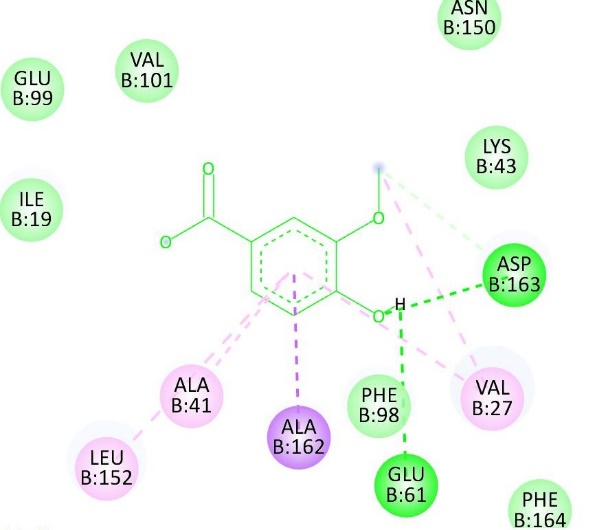 |

Table 5S. Interaction of antidiabetic protein target with the major components of *M. vulgare* extract ligands representing the binding pocket residue amino acids with distances and type of interacting bonds.

| Ligands with 4W93  antidiabetic protein | Interacting  Amino Acid  Residues | Interacting Amino Acid Residues Distance in Angstrom | Types of Bonding Interactions |
| --- | --- | --- | --- |
| Apigenin | GLN63  GLN63  GLN63  GLU233  TRP59  TRP59  TRP59  TYR62 | 2.89909  2.47918  2.76613  2.73158  5.23318  3.99862  4.16664  4.49933 | H-bond  H-bond  H-bond  H-bond  hydrophobic  π – bond  alkyl-bond  alkyl-bond |
| Biotin | ARG252  ARG421  ARG421  PRO332  SER289  ASP402  GLY334 | 1.8538  2.60317  1.90977  2.83235  2.53286  3.76845  3.63394 | H-bond  H-bond  H-bond  H-bond  H-bond  π – bond  π – bond |
| Caffeic acid | ASP197  TYR62 | 2.51614  4.35016 | H-bond  alkyl-bond |
| Catechin | ASP197  GLU233  ASP197  TRP59  TRP59  TYR62 | 2.89267  2.05207  2.51375  4.12758  4.4063  4.66836 | H-bond  H-bond  H-bond  alkyl-bond  alkyl-bond  alkyl-bond |
| Leotulin | GLN63  H - O  TRP59  TRP59  TRP59  TYR62 | 2.57725  1.98565  5.34799  4.07022  4.33105  4.33933 | H-bond  H-bond  alkyl-bond  alkyl-bond  alkyl-bond  alkyl-bond |
| Maleic acid | ARG346  ARG346  ASP317  GLN302 | 1.95887  2.12301  1.98687  1.98987 | H-bond  H-bond  H-bond  H-bond |
| Salicylic acid | ARG195  ASP197  GLU233  ASP197  TYR62 | 2.19493  2.10143  2.7312  2.46405  4.49881 | H-bond  H-bond  H-bond  H-bond  alkyl-bond |
| Vanillic acid | ASP197  ASP197  GLU233  TYR62  ALA198  LEU162 | 2.32488  2.48991  3.73678  4.50261  3.75374  4.98117 | H-bond  H-bond  π – bond  alkyl-bond  π – bond  alkyl-bond |

Table 6S. Interaction of antidiabetic protein target with the major components of *M. vulgare* extract ligands showing 3D and 2D structural view.

| **Ligands with 4W93** | **3D Amino Acid Interactions View** | **2D Amino Acid Interactions View** |
| --- | --- | --- |
| **Apigenin** | 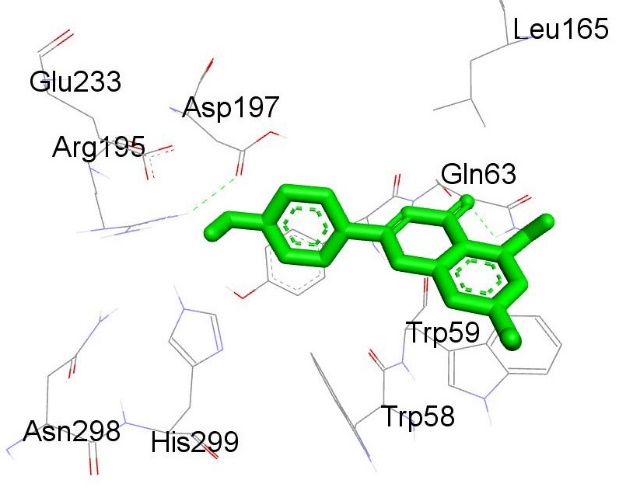 | 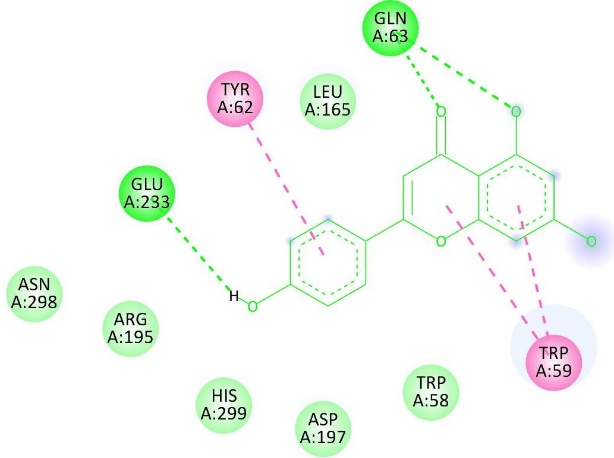 |
| **Biotin** | 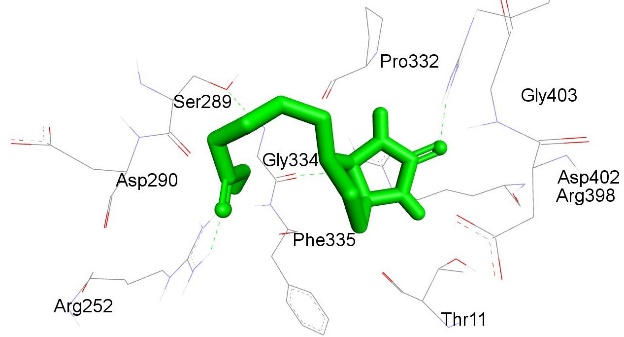 | 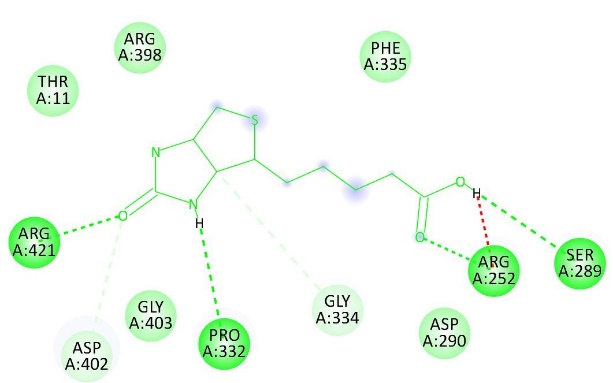 |
| **Caffeic acid** | 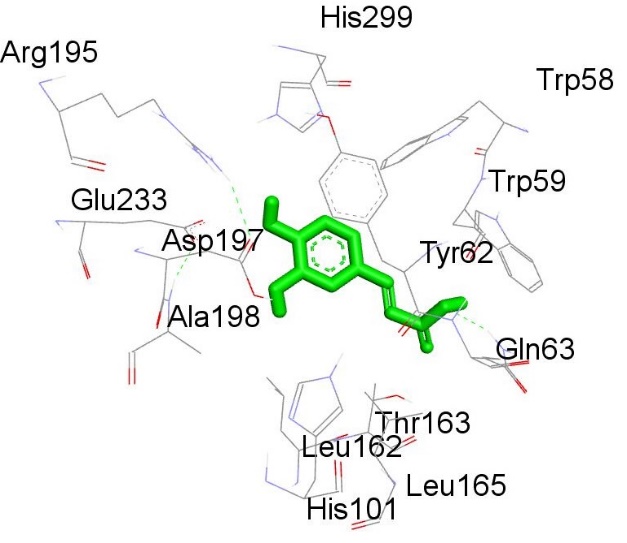 | 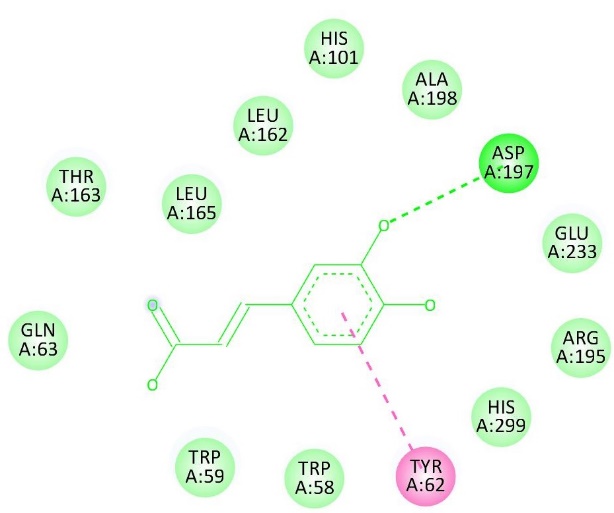 |
| **Catechin** | 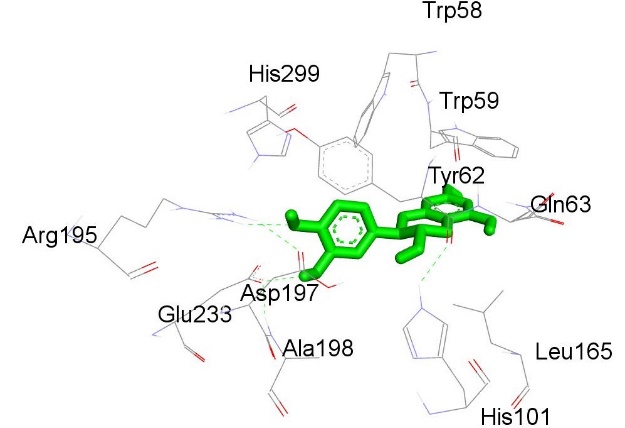 | 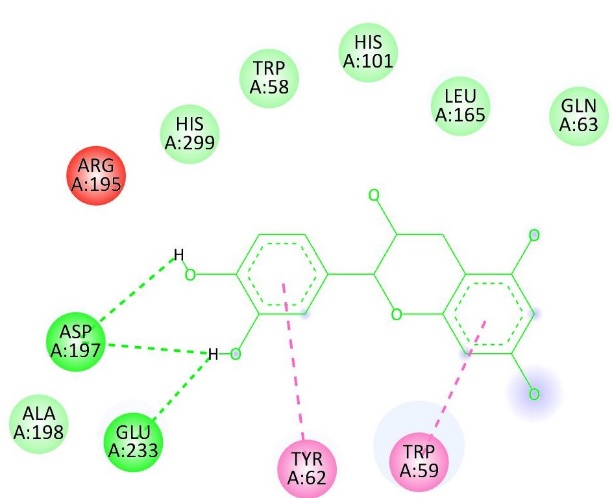 |
| **Leotulin** | 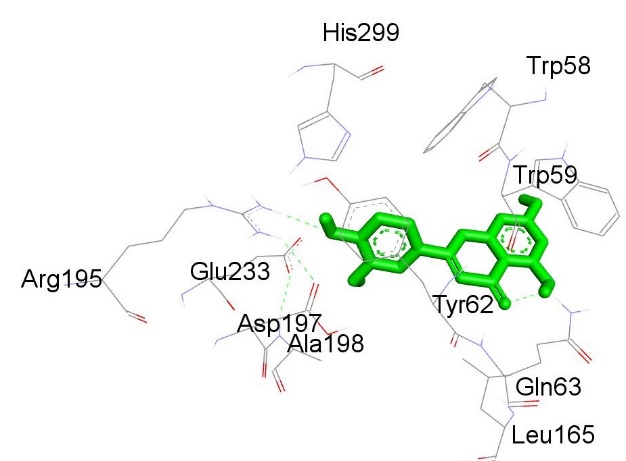 | 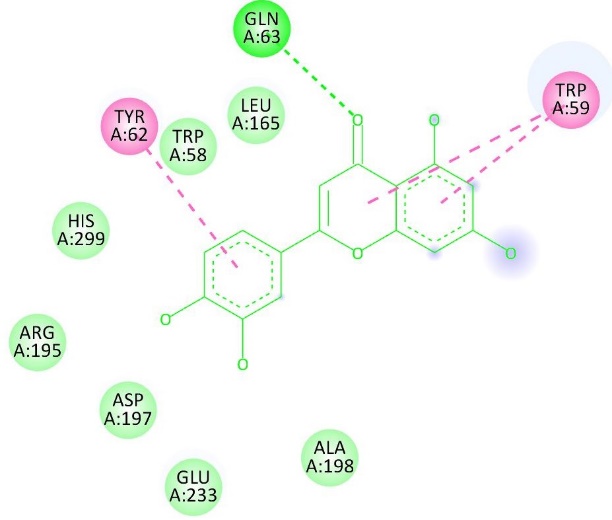 |
| **Maleic acid** | 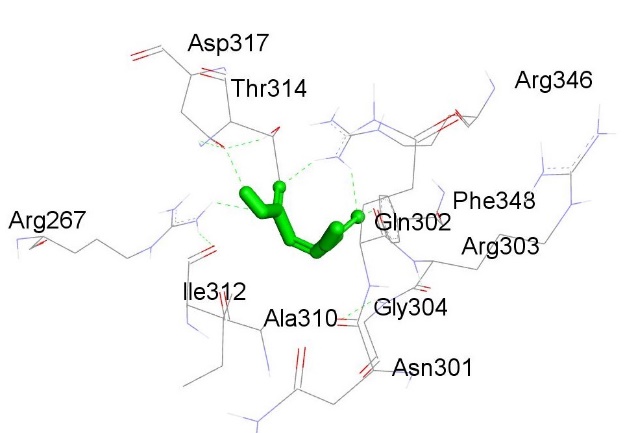 | 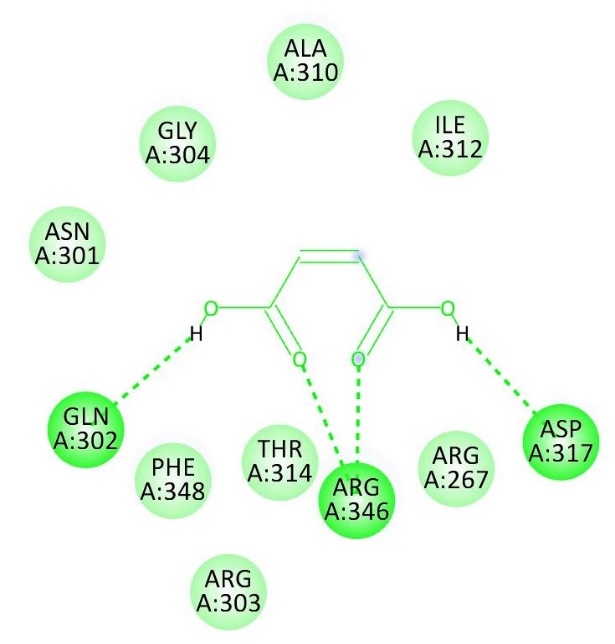 |
| **Salicylic acid** | 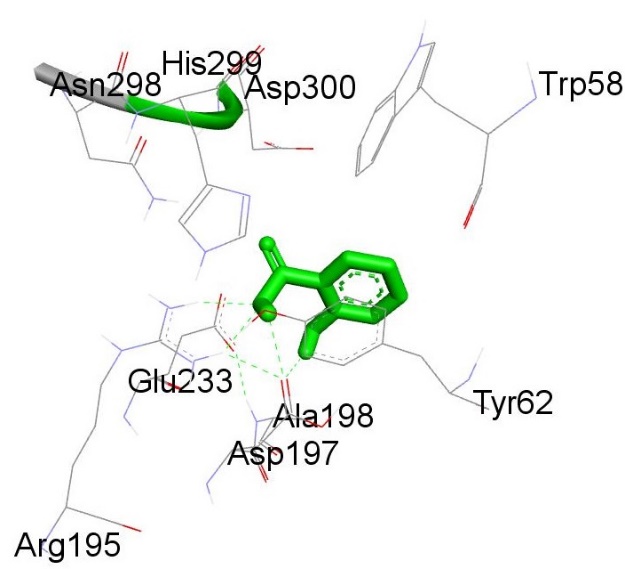 | 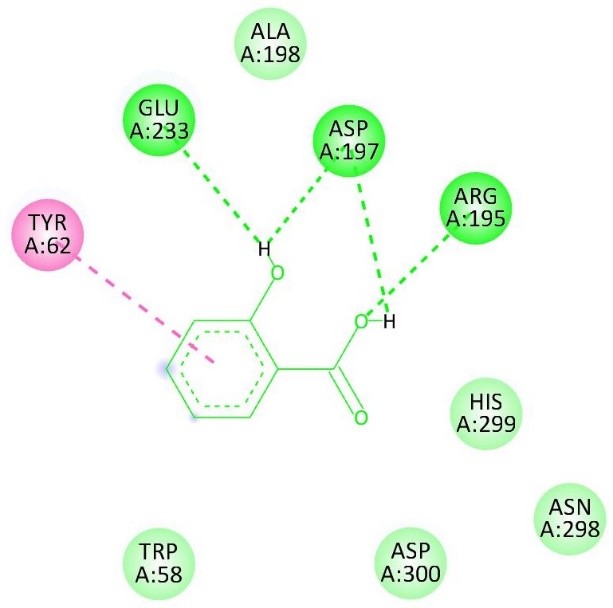 |
| **Vanillic acid** | 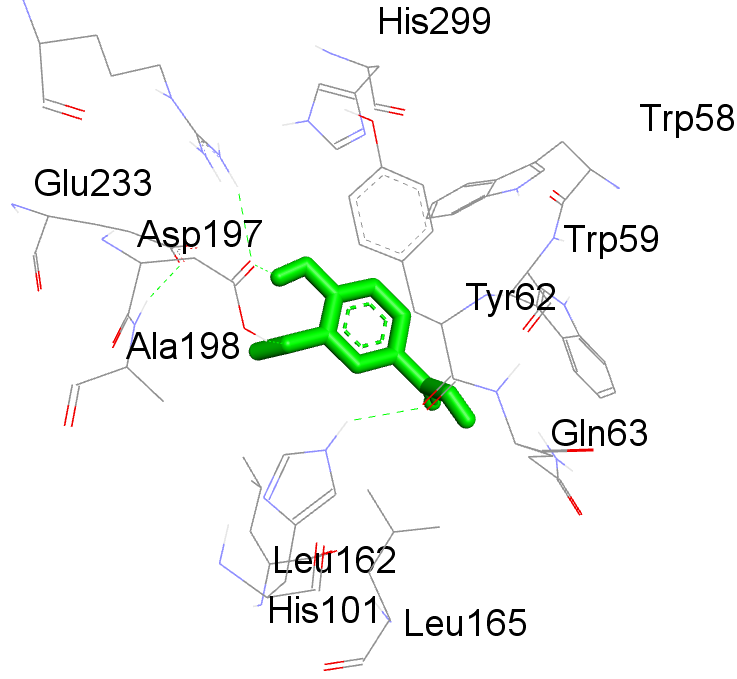 | 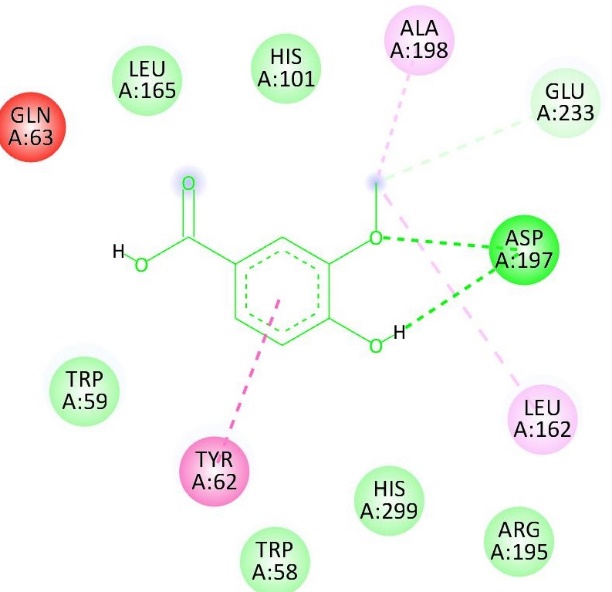 |

Table 7S. Interaction of antimicrobial protein target with the major components of *M. vulgare* extract ligands representing the binding pocket residue amino acids with distances and type of interacting bonds.

| Ligands with 1AJ6  antimicrobial protein | Interacting Amino Acid Residues | Interacting Amino Acid Residues Distance in Angstrom | Types of Bonding Interactions |
| --- | --- | --- | --- |
| Apigenin | H-O  GLY113  ILE186  HIS38  ARG190  LYS189  ARG190 | 1.8454  2.21433  3.74955  4.53453  5.18189  5.03933  5.34399 | H-bond  H-bond  π – bond  alkyl-bond  hydrophobic  hydrophobic  hydrophobic |
| Biotin | GLY77  THR165  ASP73  ALA100  ILE78 | 2.433  2.58821  2.32752  2.85643  5.36772 | H-bond  H-bond  H-bond  H-bond  hydrophobic |
| Caffeic acid | THR165  ASP73  ASP73  GLY77  GLU50  ILE78 | 2.27111  2.05796  2.77092  2.04553  3.44925  3.81553 | H-bond  H-bond  H-bond  H-bond  π – bond  π – bond |
| Catechin | ARG76  ARG76  GLU50  THR165  ALA47 | 2.61913  2.75786  2.71176  3.91402  5.25295 | H-bond  H-bond  H-bond  π – bond  hydrophobic |
| Leotulin | ARG76  ARG76  GLU50  GLU50  ASN46  THR165  ILE78  ALA47 | 2.58812  2.88023  2.56108  3.52198  3.52619  3.82137  5.05026  5.0265 | H-bond  H-bond  H-bond  π – bond  π – bond  π – bond  hydrophobic  hydrophobic |
| Maleic acid | HIS99  ALA100 | 2.67941  2.33271 | H-bond  H-bond |
| Salicylic acid | ASP73  VAL120  VAL167 | 2.39911  5.20708  5.39453 | H-bond  hydrophobic  hydrophobic |
| Vanillic acid | THR165  ASP73  VAL71  ASP73  THR165  ALA47  VAL43  VAL71  VAL167  ILE78 | 2.75219  1.97698  3.58663  3.70896  3.89515  3.95965  5.26795  4.54824  5.01758  5.36435 | H-bond  H-bond  π – bond  π – bond  π – bond  π – bond  hydrophobic  alkyl-bond  hydrophobic  hydrophobic |

Table 8S. Interaction of antimicrobial protein target with the major components of *M. vulgare* extract ligands showing 3D and 2D structural view.

| **Ligands with 1AJ6** | **3D Amino Acid Interactions View** | **2D Amino Acid Interactions View** |
| --- | --- | --- |
| **Apigenin** | 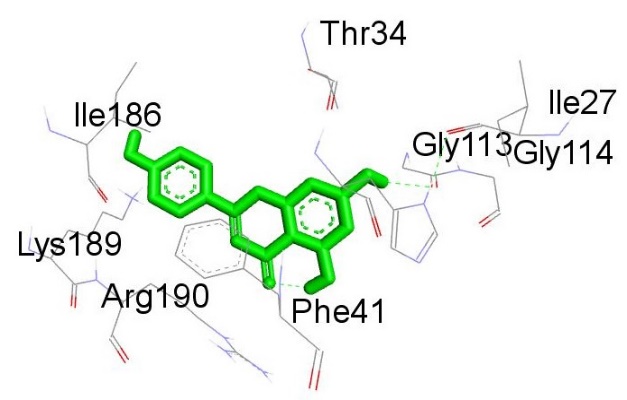 | 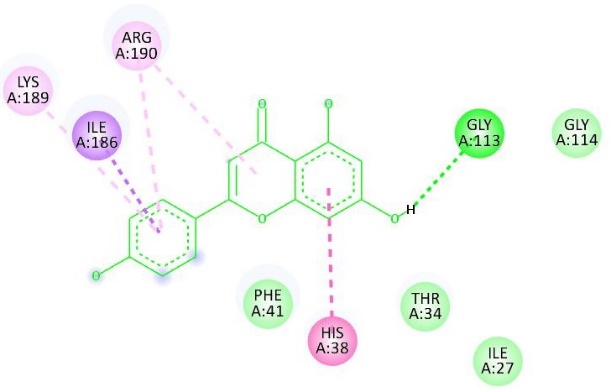 |
| **Biotin** | 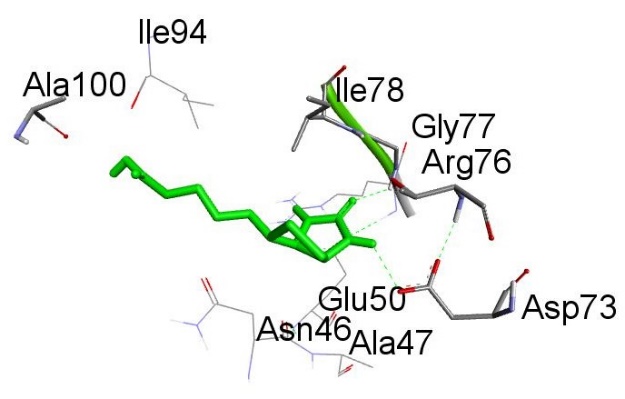 | 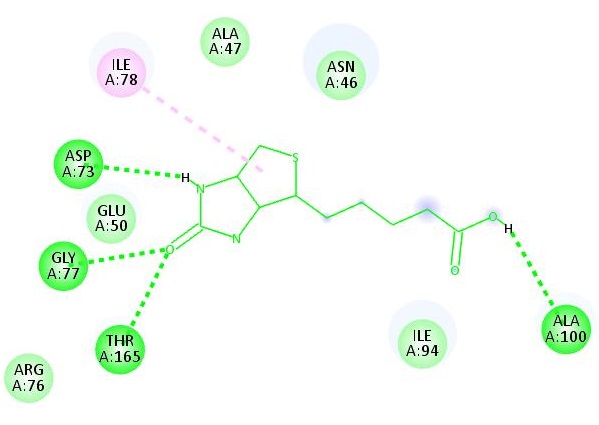 |
| **Caffeic acid** | 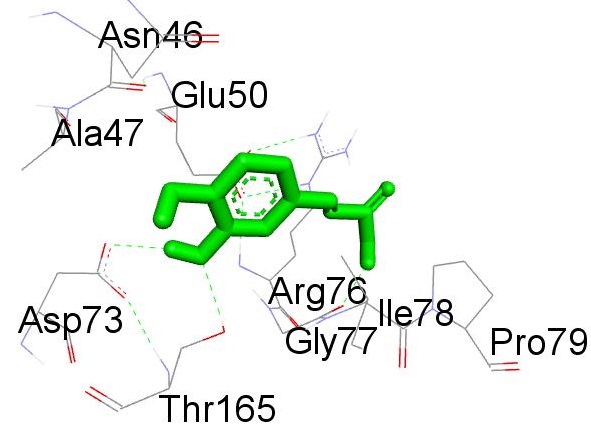 | 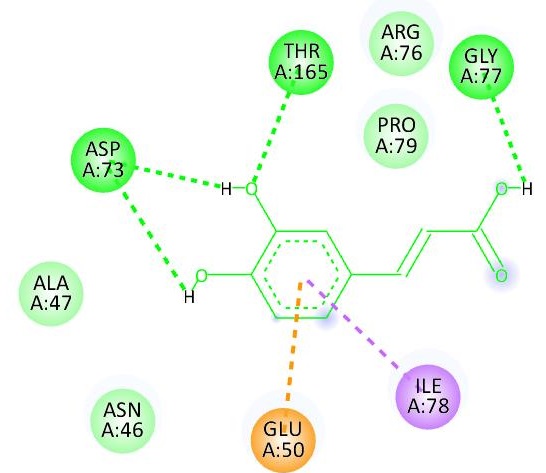 |
| **Catechin** | 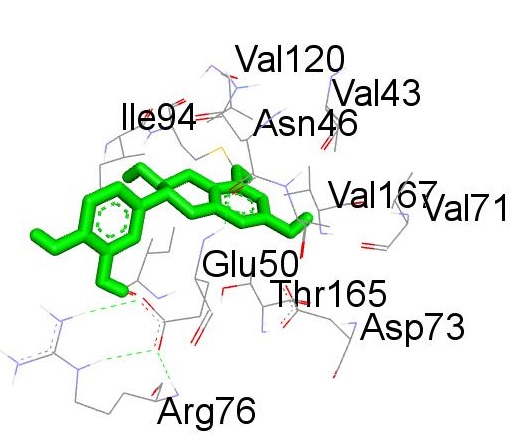 | 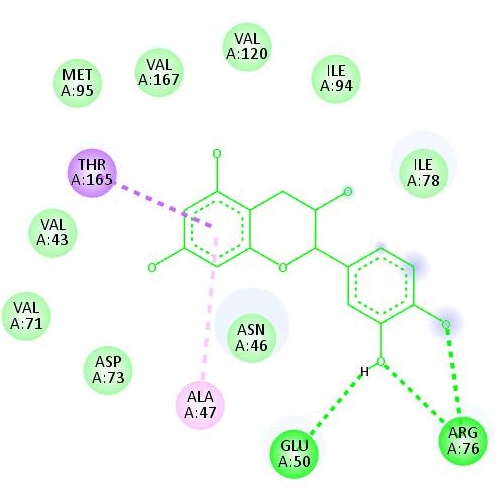 |
| **Leotulin** | 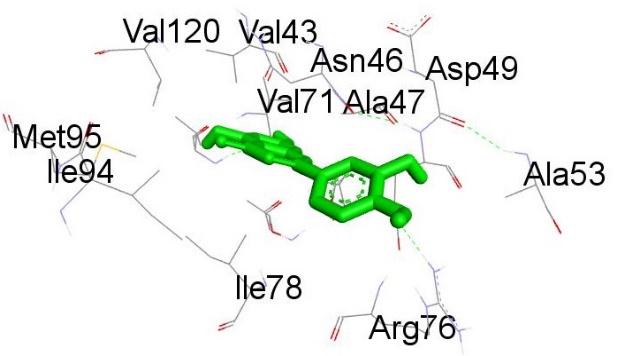 | 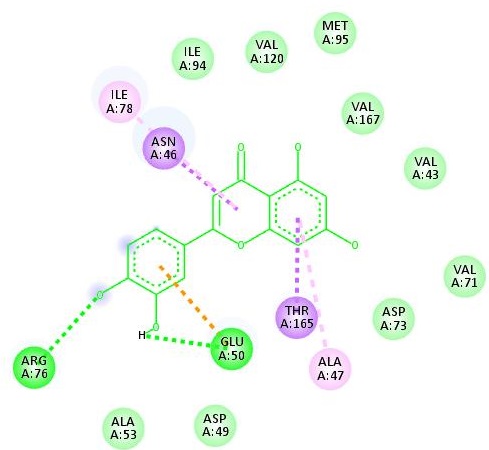 |
| **Maleic acid** | 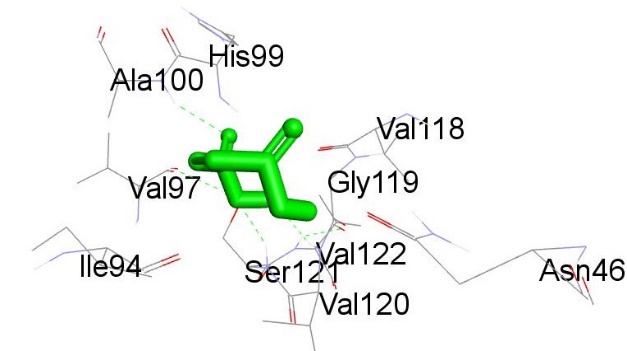 | 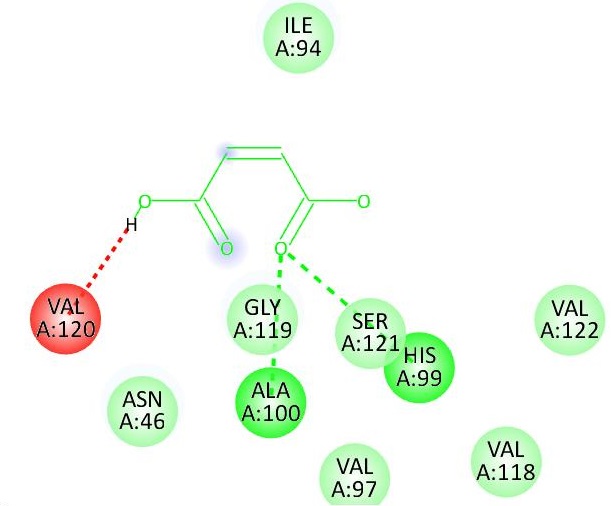 |
| **Salicylic acid** | 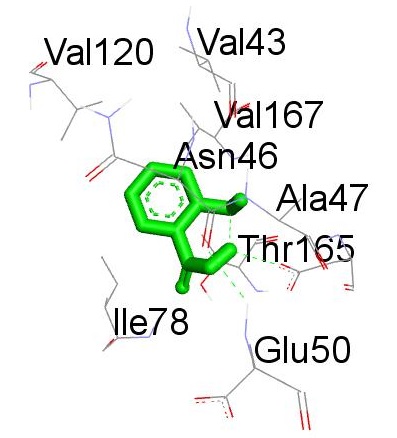 | 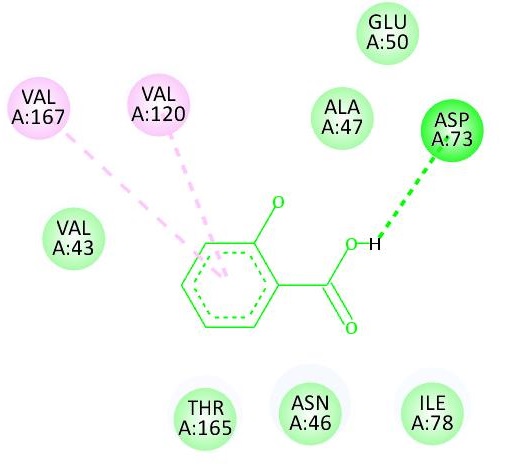 |
| **Vanillic acid** | 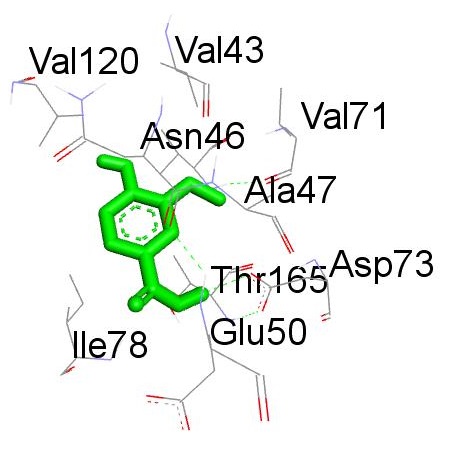 | 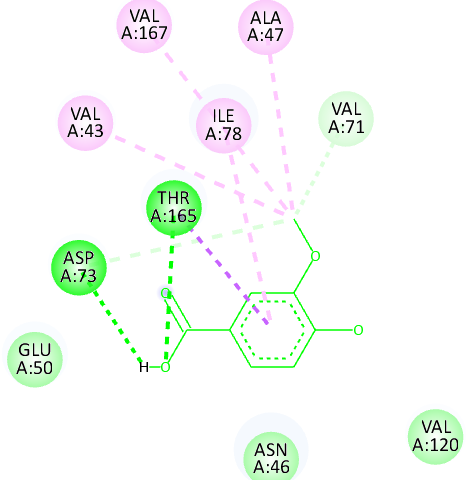 |
